# Molecular Photovoltaics in Nanoscale Dimension

**DOI:** 10.3390/ijms12010173

**Published:** 2011-01-05

**Authors:** Vladimir Burtman, Alexander Zelichonok, Andrei V. Pakoulev

**Affiliations:** 1 Department of Geology and Geophysics, University of Utah, 115 South 1460 East, Room 383, Salt Lake City, UT 84112, USA; 2 Department of Physics and Astronomy, University of Utah, 115 South 1400 East, Salt Lake City, UT 84112, USA; 3 The Institute of Chemistry, The Hebrew University of Jerusalem, Givat Ram, Jerusalem 91904, Israel; E-Mail: alex.zelichenok@ofilsystems.com; 4 Department of Chemistry, University of Wisconsin-Madison, 1101 University Ave., Madison, WI 53706, USA; E-Mail: pakoulev@chem.wisc.edu

**Keywords:** molecular photovoltaics, self-assembly devices, transport in molecular nanoscale systems, polarons in organic media

## Abstract

This review focuses on the intrinsic charge transport in organic photovoltaic (PVC) devices and field-effect transistors (SAM-OFETs) fabricated by vapor phase molecular self-assembly (VP-SAM) method. The dynamics of charge transport are determined and used to clarify a transport mechanism. The 1,4,5,8-naphthalene-tetracarboxylic diphenylimide (NTCDI) SAM devices provide a useful tool to study the fundamentals of polaronic transport at organic surfaces and to discuss the performance of organic photovoltaic devices in nanoscale. Time-resolved photovoltaic studies allow us to separate the charge annihilation kinetics in the conductive NTCDI channel from the overall charge kinetic in a SAM-OFET device. It has been demonstrated that tuning of the type of conductivity in NTCDI SAM-OFET devices is possible by changing Si substrate doping. Our study of the polaron charge transfer in organic materials proposes that a cation-radical exchange (redox) mechanism is the major transport mechanism in the studied SAM-PVC devices. The role and contribution of the transport through delocalized states of redox active surface molecular aggregates of NTCDI are exposed and investigated. This example of technological development is used to highlight the significance of future technological development of nanotechnologies and to appreciate a structure-property paradigm in organic nanostructures.

## 1. Introduction

### 1.1. Transport in Organic Semiconductors: Achievements vs. Problems

The invention of an organic donor-acceptor structural analog of a silicon p-n junction in 1974 [1] marked the beginning of the intensive research in the field of molecular electronic devices. As a part of the semiconductor research area, this field has traditionally been closely related to applications, and is unique in the synergy between device development and fundamental science. The emergence of new approaches for molecular engineering is a vital requirement for progress in molecular electronics and should eventually lead to molecular devices with pre-designed properties that may compete with silicon technologies, especially due to the small size involved. In the last years a number of different experimental strategies have been used to fabricate organic devices and to study electron transport in these devices. Despite the very impressive results in device fabrication and initial insight into device transport, the new field also brought many open questions [2,3]. One of the comprehensive analyses of this field could be found in a review by Frisbie *et al.* [4]. In this review we will focus on kinetic studies in organic structures with a pre-defined epitaxial order and on the development of adequate analytical tools to analyze charge transport in these structures.

A lack of basic understanding of structure-property relationships in organic solids results in a knowledge gap that impedes rational efforts to design organic semiconductors with further enhancements in electrical performance. One of the major obstacles for achieving efficient organic devices is the low charge mobility in most organic materials. This is an acute problem, since carrier mobilities in disordered organic semiconductor films are several orders of magnitude smaller than in inorganic semiconductors. In contrast, carrier mobility in organics could be dramatically enhanced in ordered structures, such as spontaneously ordered regio-regular polythiophene (RR-P3HT) films. However, the inherent problem of such ordered systems is the interchain coupling. The charge-transfer probability between charged polarons and bipolarons belonging to neighboring chains [5], along with high electron-phonon coupling and the Peierls transition, leads to charge trapping [6]. The strong coupling could result in low mobility and even induce pseudo-gaps [7]. In addition, disordered thin films of small organic-molecule-based electronic devices have considerable charge quenching due to carrier trapping at defects, and charge recombination at grain boundaries and interfaces that result in low electron mobility. The scope of questions about transport in SAM systems provides a host of other unanswered issues, inherent in traditional device physics, including the following: What is the role of order-disorder interplay in search for good mobilities for organic PVC? In particular, how is crystalline or partially crystalline ordering involved in the transport mechanism in organic solids? What is an impact of the thin film technology on device properties? What are the structural and electronic factors that facilitate or impede transport in organic semiconductor films? Are there particular molecular structures and crystal packing motifs that are especially favorable for transport? What is the role of film quality, defects and grain boundaries? Most of these unanswered questions are a direct consequence of the lack of basic knowledge in transport mechanisms in organic semiconductors.

The other challenge in organic PVC device fabrication is preparation of efficient n-type and p-type conductive channel. Relatively fewer n-type materials have also been reported, including C_60_ and its derivatives [8,9], oliothiophene and its derivatives [10], copper hexadecafluoro-phthalocyanine [10], naphthalene diimides, and perylene diimides [11–14]. However, some organic n-channel semiconductor materials cannot operate in air due to electron trapping by oxygen and carrier injection issues [15]. The approaches to achieve n-channel materials with air stability and high mobility have been reported by the incorporation with strong electron-withdrawing groups, such as -CN and -F [16–18]. Currently, the highest mobility of n-type organic semiconductor is obtained from a thiazole oligomer derivative (1.83 cm^2^ V^−1^ s^−1^) measured in a vacuum environment [19] or from a perylene diimide derivative with a mobility of 0.64 cm^2^ V^−1^ s^−1^ measured in air. In this review we discuss an alternative approach to achieve the desirable conductivity in SAM material by changing the type of silicon that was used as a template for the same SAM layer.

### 1.2. Analytical Tools to Study Molecular Photovoltaic Device

Lack of structure-property understanding is partly due to a shortage of adequate analytical tools to study molecular photovoltaic device operation. For example, at the current stage of development, mobility is still the most important characteristic in molecular photovoltaic devices. Does a molecular photovoltaic device have a special structure, special organic-metal interface or a peculiar charge transport mechanism? Is it just a mobility in organics, or should the whole molecular photovoltaic device structure be optimized? The situation is complicated by the strong dependence of the current through the molecular junction on the nature of the chemical bond to the electrodes [20]. Chemically bounded to the semiconductor, molecules are likely to affect the energy and density of surface states and, therefore, the semiconductor band bending [21]. Furthermore, the presence of a dipolar layer at the semiconductor/metal interface can affect the barrier for electron transport inside the semiconductor [22,23]. It is clear that molecular photovoltaic experimental results should be analyzed in terms of multiple mechanisms, and it is crucial to establish whether this is justified or if, in doing so, new mechanisms are ignored. It would be also very helpful to study independently charge injection into- and charge transport in the organic channel. However, the methods for observation and evaluation of such kinetics in organic devices at the component level have yet to be developed.

Current state of the art employs varieties of Kelvin probe-derived techniques that are widely used to determine electron work function and bend banding in organic-inorganic heterostructures. UV photoemission and inverse photoemission spectroscopes (UPS, IPES) are used to determine the position of the highest occupied and lowest unoccupied molecular orbitals (HOMO and LUMO, respectively). X-ray photoelectron spectroscopy (XPS) measurements served to estimate band bending. Comparison of these techniques with measured *I*–*V* characteristics enables one to determine mechanism(s), which control charge transfer in molecular diodes. These methods are static, since probing interfaces in time-scale compared to interface charge transfer is very challenging. Fast spectroscopy was applied to clarify dynamic of charge transfer in organic polymers, polymer-fullerenes and hybrid single-wall-carbon-nanotube structures and even in hybrid structures like dye-sensitized photovoltaic cells. Nevertheless, direct observation of complete dynamic of charge transfer in the interface is still far from the scope of these methods. A relatively simple and sensitive photocurrent method to probe transport mechanisms in organic ordered system in time resolved regime is introduced in this review. This exemplify requirement in future methodology development to analyze the transport mechanism in organic devices.

### 1.3. Self-Assembly (SAM) and SAM Molecular Photovoltaic Devices

Molecular self-assembly systems have an advantage over other organic systems in highly order structure that enable detailed study of the structural-property relationship in photovoltaic devices. The way to achieve epitaxial control in organic devices is not trivial too. The transport mechanism in low-dimensional organic structures is intimately related to the ordering and dimensionality of the underlying electronic system, which may transform with the molecular packing [24]. Therefore, a systematic research in organic semiconducting materials and structures with controlled order is a pre-requirement for efficient PVC fabrication. This fine structural tuning is a very challenging goal since tunable organic epitaxial structures have not been studied at all, in contrast to inorganic semiconductors. We explored ordered self assembled monolayer (SAM) structures of small molecules with the potential of high charge mobility and low intermolecular interaction [25].

SAMs [26–28] are ordered assemblies of functional molecules, which are formed spontaneously on an appropriate surface and are typically used to modify surface properties of materials. The tendency of certain types of molecules to spontaneously form assemblies arises from their characteristic, amphiphilic chemical structure. Self-assembling molecules are typically composed of two groups: a head group which has high affinity for an appropriate surface and a tail group with a high affinity for similar tail groups of other, neighboring molecules. When such molecules are brought in contact with the appropriate surface, the head groups of the molecules will physisorb or form a chemical bond to the surface (chemisorptions). As the density of a SAM increases, the tail groups of the molecules on the surface will come closer and start to interact, typically giving rise to the order within the self-assembled monolayer. Often the molecules also contain a functional end group which allows further physical or chemical fictionalization. Both the self-assembling and self-ordering properties of SAMs, combined with the presence of this functional end group and with the SAMs stability (due to the surface bond) are the keys to a wide range of possibilities and applications such as surface engineering and surface modification for controlling adhesion, corrosion, lubrication, (bio)chemical sensing and mimicking, *etc.* By choosing precursors with suitable physicochemical properties, it is possible to exert a fine control on the formation processes in order to obtain complex architectures. Clearly, when it comes to designing a synthesis strategy for a material the most important tool is the knowledge of the chemistry of the building units. SAM is a process that is easily influenced by external parameters. This can make synthesis more problematic due to the many free parameters that require control. On the other hand, this has the exciting advantage that a large variety of shapes and functions on many length scales can be obtained [1].

### 1.4. Charge Mobility in Naphthalene-Derivative (NTCDI) Thick Film and Self-Assembled (SAM) Devices

NTCDI-derived compounds were already tried in organic devices, including thin and thick film OFETs [29–32]. The highest mobility of solution-processed n-type semiconductors were achieved from quaterthiophene [33] (0.2 cm^2^ V^−1^ s^−1^) measured in a vacuum and from naphthalene diimide (NTCDI) with a long-chain fluorinated alkyl group (0.01 cm^2^ V^−1^ s^−1^) solution-processed in air by Katz *et al.* [30]. The highest n-channel mobilities of these devices, which were based on NTCDI derivatives were calculated to be about 1.2 × 10^−3^ cm^2^ V^−1^ s^−1^ [34]. Direct fabrication of ordered organic structures as SAM and there incorporation in molecular device is still a challenging task, and only few reports are available. Several known and new SAM deposition and characterization techniques were explored and optimized to obtain the required, qualitative SAMs for use in organic thin-film transistors [35]. Various SAMs were then applied to different interfaces in SAM-devices, which allowed a better control of the morphology and properties of the organic semiconducting material. This resulted in a significant improvement of the electrical performance of the organic transistors and even enabled a new way to pattern the organic semiconductor. Alternatively, using a technique called “molecular layer epitaxy” [36,37], multilayer structures with 2D π-stacking and semiconducting properties have been obtained. This structures have been applied in organic light emission diodes (SAM-OLEDs), and in SAM-OFETs fabrication [38,39]. A room-temperature electron mobility as high as 90 cm^2^ V^−1^ s^−1^ was reported using MLE [40]. The MLE technology is a vapor-phase oriented technique that allows the buildup of organic heterostructures via epitaxial growth of subsequent layers by interlayer covalent bonding. Vapor-phase self-assembly protocol for device fabrication introduced below is a modification of MLE approach used for new generation of SAM-OFETs. The same approach was used for a fabrication of SAM photovoltaic devises (SAM-PVC) to study photophysics of charge transfer phenomena in NTCDI channel and to model charge transport in NTCDI SAM-OFETs.

### 1.5. Transport in Naphthalene-Derivative (NTCDI) Self-Assembled (SAM) Devices

This review is focused on self-assembled monolayers of small organic molecules with extended π-electron system, NTCDI, containing two benzene rings, four carboxyl groups and two anhydrides. These two anhydrides are used to connect NTCDI molecules to substrate by chemical imide bonds (out-of-plane bonding), while two adjacent NTCDI molecules held together in a solid monolayers by van der Waals forces (in-plane bonding). Weak van der Waals bonding in in-plane direction and weak intermolecular overlap of electronic orbitals lead to the narrow electronic bands (typical bandwidth ~0.1 eV is two orders of magnitude smaller than that in silicon) and the low mobility of charge carriers (μ ≈ 1–10 cm^2^ V^−1^ s^−1^ at room temperature). Anisotropy of the transfer integrals between adjacent molecules reflects the low symmetry of the molecular packing. It is believed that the most adequate description of charge transport in these organic semiconductors is based on the concept of polarons, molecular states with a correlation between the electronic and lattice degrees of freedom at the scale of the order of the lattice constant [41–44].

After several decades of intensive research, our basic understanding of charge transport in small-molecule organic semiconductors remains limited [45]. The complexity of transport phenomena in these systems is caused by the polaronic nature of charge carriers and the strong interaction of the polarons with defects [45]. Organic PVCs are aimed to be used at room temperature. Therefore, the high-temperature polaronic transport should be developed. At room temperature, which is typically comparable to or even higher than the characteristic phonon energies, lattice vibrations might become sufficiently strong to destroy the translational symmetry of the lattice. In this regime the fluctuation amplitude of the transfer integral becomes of the same order of magnitude as its average value [46]. The band description breaks down, and a crossover from the transport in delocalized states to the incoherent hopping between localized states is predicted with temperature increase [47–53]. While time-of-flight experiments demonstrated that a possibility of the intrinsic (not limited by static disorder) charge transport can be realized in the bulk of these crystals [54], the mechanism of the charge transport on the surface of organic semiconductors is less studied [55–57]. It is clear that in SAM-PVC devices light-induced charges should propagate along the interface between a SAM and a gate dielectric, which are true surface conditions. The differences in transport mechanism for surface and bulk media include: (i) the density of carriers, which in transport experiments can exceed that in bulk measurements by many orders of magnitude, approaching the regime when the intercharge distance becomes comparable with the size of polarons [58] (ii) motion of charge carriers in the light-induced conduction channel may be affected by the polarization of the interface [59], and (iii) molecular packing on the surface can also be different from that in the bulk. This review will expand the study of correlation between dynamic characteristics (time-of-flight measurements) and charge mobility in bulk crystals [55] to the case of the SAM system.

Exploration of the polaronic transport on organic surfaces is crucial for a better understanding of fundamental processes that determine operation and ultimate performance of organic electronic devices. Fundamental research has been hampered by the lack of a proper tool for exploring the polaronic transport on surfaces of organic semiconductors. Over the past two decades a large effort in the development of PVCs and OFETs has resulted in improvement of the characteristics of these devices [60]. However, even in the best SAM devices, charge transport is still dominated by the presence of structural defects and chemical impurities.

### 1.6. Background of SAM Fabrication Technology

The vapor phase approach to molecular self-assembly (VP-SAM) was proposed in 1996. The VP-SAM deposition system was built by Vacuum System and Technology (VST) Co. in 1998. In following two years the VP-SAM fabrication technology was developed. The capability of this technology to grow organic superlattices in the monolayer-by-monolayer fashion was demonstrated and VP-SAM kinetic was studied. Organic light emitted diodes (OLED’s) and first OFET were fabricated and tested in 1998–2000. It was realized even at very early stage of this project that in-plane order in VP-SAM structures is extremely interesting. We anticipated from the very beginning that in-plane structural arrangement should define the new properties in (VP-SAM)-derived devices. However the full complexity of transport phenomena in VP-SAM structures was not appreciated at that time.

A VP-SAM technology was designated as molecular layer epitaxy technology or MLE in 1998. High electron mobility in OFET devices was, probably, the largest success of MLE technology. The electron mobility was reported as high as 90 cm^2^ V^−1^ s^−1^ [39,40]. These results were accomplished in first set of experiments, which were not reproduced after several researchers left the research team. The reasons of anomaly high electron mobility and transport mechanism were not studied at that time. The MLE technology was commercialized at 2000. MLE technology was developed by Nanolayer Startup Company, with a goal to peruse the OFET applications of MLE method. The commercialization of MLE technology was not successful. Since that time, the MLE method seems to be in disrepute. The authors of this review were not affiliated with Nanolayer. In our late studies we turned back to initial VP-SAM acronym instead of MLE to distinguish our academic research from the commercial spin-off of this technology.

### 1.7. The Scope of This Review

In this paper, we present a brief overview of the experimental results obtained with NTCDI SAM-PVCs over the last years. Because we focus on the physics of electronic processes in these devices, many surface chemistry oriented issues will not be discussed here; for details on SAM fabrication and characterization we refer the reader to a bible of self-assembling by A. Ulman [26,27]. This much-cited, definitive text has become the standard resource on the SAM topic worldwide. In the Materials and Methods section we briefly describe the SAM growth, OFET and organic PVC fabrication techniques and measurement techniques. The Results section focuses on the observation of intrinsic polaronic transport in SAM devices. Electronic mechanisms of charge transport are discussed in the Discussion section. Structural studies and polaron transport in the vapor phase molecular self assembled (VP-SAM) structures were studied in more details using model compounds. The Conclusion section outlines several fundamental problems that became experimentally reachable due to the development of SAM-PVCs technology.

## 2. Materials and Methods

Several variations of SAM devices were studied for the same 1,4,5,8-Naphthalene-tetracarboxylic diphenylimide (NTCDI) SAM system to evaluate a transport mechanism in SAM-PVCs. These SAM devices are: (i) SAM-OFETs and (ii) hybrid self-assembled photovoltaic cells (SAM-PVC). SAM-PVC devices were fabricated on p-and n-Si substrate.

### 2.1. Preparation of Organic Films

**Figure 1:**
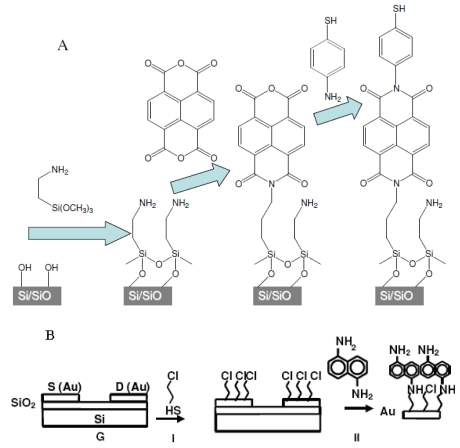
Fabrication of SAM devices. (**A**) The three-step assembly of monomolecular films of NTCDI on oxides Si and Si_3_N_4_ substrates (conductive channel at SAM-OFET and at SAM-PVC). Step (a): Thick, impermeable to light wafers of Si(100) having 20 Å of SiO_2_ coating and glass slides were cleaned and functionalized with amino groups. Step (b): NTCDA (1,4,5,8-naphthalene tetracarboxylic anhydride) evaporated at 110 °C and reacted with the NH2-functionalized surface for 45 min in a Bell Jarr chamber at 10^−5^ Torr. Step (c): A layer of 4-aminophenylthiol was added within 20 min. (**B**) The two-step assembly of amino-coated template layer on Au electrodes used only for SAM-OFET source and drain. Step (I): 3 mM solution of 3-chloro-1-propanethiol (Cl-Pr-SH) in ethanol was spin-coated on the OFET substrate to form AuS bonds. Step (II): a 5 mM solution of 1,5-diaminonaphtalene (DAN) in ethanol was spin coated on the surface followed by annealing at 100 °C for 1 h to form imine bonds with template layer. SAM of NTCDI was continued from step b of (**A**), following assembly of aminated template layer on Au.

Step-A: SAM on SiO_2_ and glass for SAM-OFET conductive channels and SAM-PVCs (Figure 1A). Thick, impermeable to light wafers of n-type (500 Ω·cm, Virginia Semiconductors) Si(100) having 20 Å of SiO_2_ coating and glass slides were cleaned and functionalized with amino groups (step a), Figure 1A, as described in [61]. In the experiment, which was aimed to invert the conductivity type of NTCDI channel, a p-type Si(100) having 20 Å of SiO_2_ (100 Ω·cm, Virginia Semiconductors) was used in addition to the n-type silicon wafers. NTCDA (1,4,5,8-naphthalene tetracarboxylic anhydride) evaporated at 110 °C and reacted with the NH_2_-functionalized surface for 45 min in a Bell Jarr chamber at 10^−5^ Torr (step b).

The product of assembly lacks the amino group preventing formation of the second layer. The substrate was kept on a heated sample holder (180 °C) preventing physadsorption of the precursor. Vacuum deposition modified over 60% of the surface amino groups [62]. A layer of 4-aminophenylthiol was added within 20 min (step c). This reaction tops the surface with SH-groups reactive toward metals [63]. The chip was gently rinsed with 2-propanol and heated at 80 °C for 1 h. A silver contact was placed on top of the films by placing a drop of colloidal silver in acetonitrile and allowing the solvent to evaporate; the area or the contact between the silver and the coated silicone was ~3 mm^2^.

Step-B: SAM on Au electrodes for SAM-OFET source and drain (Figure 1B). First, a 3 mM solution of 3-chloro-1-propanethiol (Cl-Pr-SH) in ethanol was spin-coated on the OFET substrate to form AuS bonds (I in Figure 1B). The substrates were held under Ar flow for 20 min after spin-coating and after that the substrates were rinsed with isopropyl alcohol and heated at 100 °C for 1 h. Next, a 5 mM solution of 1,5-diaminonaphtalene (DAN) in ethanol was spin coated on the surface followed by annealing at 100 °C for 1 h to form imine bonds with template layer (II in Figure 1B). The excess DAN was removed by an ethanol rinse and annealed at 100 °C for 1 h. A self-assembly route described above for oxidize surface and depicted at Figure 1A was completed SAM-OFET device fabrication. Different self-assembly of template layers on OFET conductive channel, following step a in Figure 1A, and electrodes, following step (II) in Figure 1B, ensure formation of unified NTCDI layer across SAM-OFET surface and device integrity.

In addition, few samples were prepared for SAM-PVC devices. Thick (~300 nm) films of NTCDA and C_6_-NTCDI were prepared by physadsorption of NTCDA on the cold functionalized surface of silicon. Sparse NTCDI films were obtained from thick films of NTCDA by heat desorption of the excess of NTCDA followed by step c. Step b was not employed in SAM-PVC devices.

### 2.2. Characterization of the NTCDI Monolayer

A step-by-step buildup of the layers was characterized by contact angle (CA) changes, IR spectroscopy, XPS, and UV-vis spectroscopy. The results agree well with previous characterization 62, 63 of NTCDI SAM monolayers and are briefly summarized below. After step a, CA changed from 17° to 45°, and the appearance of the 3200 cm^−1^ alkylamine IR peak was observed. The N(1s) XPS core level spectra showed a predominantly 398.8 eV (85%) peak on the amine surface because of the nonprotonated NH_2_-group [64]. The minor N(1s) component at 400.6 eV is attributed to protonated NH_3_^+^ (15%). The UV-vis spectrum of the film grown on the glass slide did not reveal any peaks. After step b, the CA changed from 45° to 92° and the 1655 cm^−1^ IR peak of the imide bond formation was detected. In accord with previous studies [65], formation of imides on the surface results in disappearance of the majority of the original peaks at 400.6 eV (29%) and 399.8 eV and formation of a new single broad N(1s) peak at 399.6 eV (67%) because of formation of imido groups with a possible presence of amido groups [66] When step b was performed on a glass slide, two peaks appeared in the UV spectrum: 360 and 390 nm; OD ≈ 0.004 and 0.006, respectively. We also observed an appearance of a new broad peak in the greenish-orange region (OD = 0.001) that characterized a formation of an in-plane ordered organic heterostructure in SAM structure. After step c, the CA changed from 92° to 60° and a new peak (236 eV) associated with HS groups was observed in the XPS spectrum. No significant changes were found in the UV-Vis and the IR spectra after step c. Variable angle spectroscopic ellipsometery (VASE, Woollam Co.) was used to verify the monolayer growth as it was reported for NTCDI SAM structure [37,62,63]. The variable angle spectroscopic ellipsometer measured spectra with 5-nm intervals in the range 300–1700 nm. The structural model for fitting *ex situ* ellipsometry data uses the data of three different incident angles: 65°, 70°, and 75°. Measured and fitted ellipsometric data for a structure containing Si/SiO_2_, siloxane matrix, NTCDI-benzene thiol exhibit molecular *c*-axis interplanar spacing are 20 Å for SiO_2_ native oxide, 3.5 Å for the siloxane matrix, 7.0 Å for NTCDI, and 6.9 Å for the benzene thiol layer.

### 2.3. SAM-OFET Electrode Configuration

**Figure 2:**
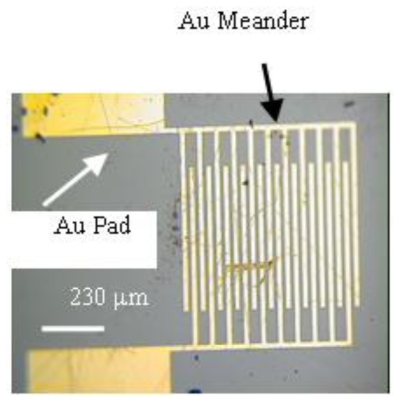
FET electrode configuration. Optical image of electrode meander before deposition.

Substrates for OFETs and tunneling devices were fabricated using reactive ion etch-grown silicon nitride (100 nm) as the insulating layer on highly doped n-type silicon, which also functioned as the gate electrode. Gold source and drain interdigitated contacts were photolithographically defined on the silicon nitride to give total channel width W_1_ = 6000 μm (width is 400 μm for 15 meander elements) and channel lengths L_1_ = 20 μm. Figure 2 shows a top view of the interdigitated finger array. The substrates were cleaned by immersing for 1 h into a solution containing H_2_O/H_2_O_2_/NH_3_ (5:1:1) while sonicating. After that, they were washed with deionized water (17.8 MΩcm) and acetone, subsequently, and heated in an oven for 30 min at 100 °C. Following fabrication step a and step b, which are describe above, indium contacts were attached to drain and source Au pads (Figure 2). Silver paste was used to connect to bottom gate electrode.

### 2.4. Determination of the Absorption and External Quantum Efficiencies in Closed-Circuit SAM-PVCs

The spectra of dense and sparse monolayers of NTCDI grown on glass slides were recorded in a Shimadzu spectrophotometer. Comparison of the films grown on Si and glass is justified by the surface titration, which shows that the density of amino groups on glass and SiO_2_ coating of Si(100) is approximately the same [62] (2–3/100 Å^2^). The EQE was calculated as a ratio of the number of electrons passed through a 500 Ω resistor to the number of photons in the light flow. The output of a Xenon lamp/monochromator assembly (8-nm bandwidth) standardized to the Oriel calibrated light source was used to illuminate the photoelement. The EQE was also measured at 532 nm by referring to nonsaturating output of CW YAG laser calibrated with a photocalorimeter.

### 2.5. Time-Resolved Photovoltaic Measurements in Closed-Circuit SAM-PVCs

**Figure 3:**
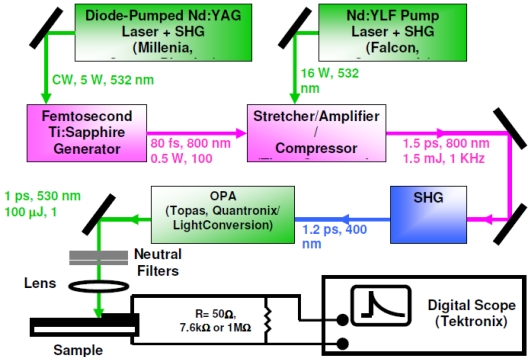
Experimental setup for a time-resolved photovoltage measurement in closed-circuit SAM-PVCs.

These were performed with a picosecond laser system consisting of a Ti: Sapphire femtosecond laser, stretcher/compressor/amplifier of picosecond laser pulse (“Titan”, Quantronix), and optical parametric amplifier of superflourescense (“TOPAS”, Qunantronix/Light Conversion). The output pulse parameters were: wavelength 532 nm, energy 10 μJ, pulse duration 1 ps, and repetition rate 1 kHz. To avoid saturation effects, the pulse energy was attenuated to a 0.1 μJ level with a beam diameter of 1 mm. The photovoltage transients were recorded by Tektronix TDS 3025 digital oscilloscope bypassed with a 7.6 kΩ resistor (Figure 3).

### 2.6. Capacitance Coupling (Open-Circuit) Measurements in SAM-PVC

To learn the charge separation we studied the formation and decay of the surface photovoltage by an auxiliary transparent electrode, which serves as a capacitive probe that picks the transient surface photovoltage. This method has been used in electronics and biophysics but is novel for the surface redox chemistry. The probe both provides the direction of the charge transfer and resolves its dynamics. Its application is independent of the optical properties of the semiconductor. Laser initiation of the reaction permits quantitative measurements of the reactions with the rate constants ~10^8^–10^3^ s^−1^. The back electron transfer is poly-disperse with the components ranging from ~10 μs and up. The measurement scheme was the same as in Figure 3, but instead of upper electrode a semi-transparent indium tin oxide electrode was used to achieve a capacitor coupling. To learn the charge separation we studied the formation and decay of the surface photovoltage by an auxiliary transparent electrode.

### 2.7. Synthesis of Naphthalene-Diimide Model Compounds

UV-Vis absorption spectra were recorded on Shimadzu’s UV-Vis-NIR 3101 PC scanning spectrophotometer. Luminescence was measured with RF-5301 PC spectrofluorimeter. IR spectra were recorded using the Bruker FT-IR spectrometer; model IFS 113v, with spectral resolution of 2 cm^−1^. Samples with 1% concentration of NTCDA precursor and model compounds (**1**–**4**) in KBr pressed powder pellets were used. The different scanning calorimetry (DSC) measurements of model compounds were done with Mettler TA 300 calorimeter at temperature rate 10 K/min. Polarization optical microscopy observations were performed on Zeiss Universal microscope equipped with a Mettler FP 52 heating stage. The colored pictures reported here were taken with transmitted light using crossed polarizers. Temperature depended XRD spectra were recorded in X-ray Searle low angle camera operating with Ni-filtered Co-Kα radiation (λ_average_ = 1.54 Å), which was further monochromated and collimated by double Francs mirrors. The X-rays were generated by an Elliot GX6 rotating anode generator operating at 35 kV and 35 mA with 100 μm focus. Samples were held in 1.5 mm glass X-ray capillaries and placed in homemade furnace, which consist on copper and Teflon cavity equipped with a triac temperature control. The temperature was measured with an iron-constant (type J) thermocouple. The diffraction pattern were reordered on imaging plates (Fuji), calibrated with cholesterol (d = 34 Å) and scanned with a He-Ne laser (Spectra-Physics) on conjunction with a homemade reader based on an Optronics (Chemford, Mass) densimeter and interfaced to an Appolo DS 3500 work station.

## 3. Results

### 3.1. NTCDI SAM OFET Devices

**Figure 4:**
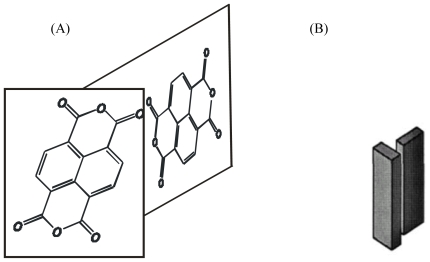
(**A**) Natural herring-bone pattern packing of NTCDA molecules; (**B**) Scheme of orthogonal NTCDI intermolecular plane configuration.

In order to have high mobility and low charge trapping in organic semiconductors, we have focused on the class of organic materials with *herring-bone* crystallographic packing. Herring-bone packing could prevent interchain quenching and, at the same time, have high electron and hole mobilities. The aromatic polycyclic structures, which are based on small molecules, and have a natural herring-bone pattern packing in the crystals [67] are promising candidates to that end. For example, one of them with the highest mobility in crystalline form (μ = 3 × 10^−3^ cm^2^ V^−1^ s^−1^), naphthalene tetracarboxylic dianhydride (NTCDA) has such herring-bone pattern (Figure 4) [68]. Moreover, it was shown that NTCDA conductance in crystalline could be enhanced by two orders of magnitude by increasing charge concentration through doping [69]. Our research showed that SAM of NTCDI molecules results in ordered structures, which have even higher electron mobility.

**Figure 5:**
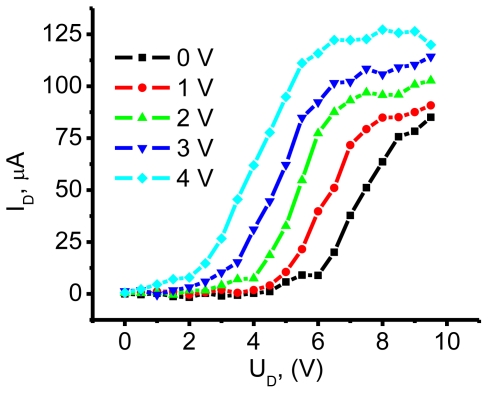
Resulting *I*_D_–*U*_D_ curves at different gate biases.

Figure 5 shows the *I*–*V* curves of a SAM-OFET device. The device exhibits saturation regime at high enough drain voltages. V_D_ of 0.5 V was required in order to obtain the drain current. The nature of this barrier will be clarified at discussion section following photocurrent studies in NTCDI hetero-structures. Detailed discussion of the ways to decrease metal-organic injection barrier in SAM-OFET is beyond the scope of our contribution. Our preliminary experiments indicate that doping of template layer by molecules carrying positive and negative charge could essentially affect this barrier. In particular, using of 0.01 μM solution of tri-phenyl amines decreases this barrier to 0.1 V without effecting essentially mobility in SAM-OFET device.

**Figure 6:**
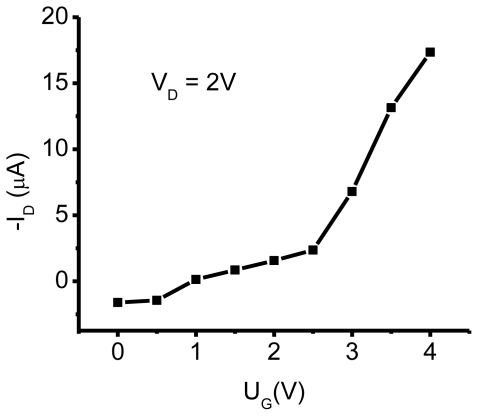
Transconductance measurements of SAM-OFET device showing n-type behavior at *V*_D_ = 2V.

The mobility of this n-type device was calculated at the saturation region using the next equation:

(1) $$\mu_{\text{e}}=\frac{\Delta I_{\text{D}}}{\Delta V_{\text{G}}}\frac{L}{W}\frac{1}{V_{\text{D}}}\frac{1}{C_{\text{OX}}}$$

where $C_{\text{OX}}=\frac{t_{\text{OX}}}{{ɛ}_{\text{S}}+{ɛ}_{0}}$ is capacity of SiO_2_ layer, *t*_OX_ is thickness of SiO_2_ layer, ɛ_S_ he static permittivity of the material and ɛ_0_ is dielectric constant, *L* and *W* are length and width of conductive NTCDI channel, *V*_D_ is drain bias, under which mobility is calculated, Δ*I*_D_ and Δ*V*_G_ are drain current and gate bias range in which mobility is calculated. Here $C_{\text{OX}}=\frac{2\times 10^{-6}}{4\times 8.86\times 10^{-14}}\left(\frac{\text{cm}}{F/\text{cm}}\right)$, *L* and *W* are 20 μm and 6000 μm, *V*_D_ is 2V (Figure 6) and ratio $\frac{\Delta I_{\text{D}}}{\Delta V_{\text{G}}}=\frac{6\times 10^{-3}}{2}\left(\frac{A}{V}\right)$. n-type mobility (μ_e_) of 31.5 cm^2^ V^−1^ s^−1^ is obtained by applying Equation 1 to the data in Figure 4 and a high on/off ratio of up to 10^9^ is obtained. We note that such a high mobility was achieved only in 10% of fabricated devices. In total, 20 SAM-OFET devices were tested. Twenty percent of the tested devices were shortened and about 35% of devices were damaged during measurements. Roughly 35% of devices have a lower mobility or exhibit properties of gated tunneling diodes. These tunneling diodes demonstrated typical Flowler-Nordhaim tunneling features [70]. Here we will discuss transport properties only of the best devices. The device reproducibility aspect will not be discussed in this chapter. This is a rather high mobility, considering the thickness of the organic semiconducting layer. The transconductance of the device is shown in Figure 6 and demonstrates the turn on voltage of the device in an n-type mode on the same device. In this device, a *V*_D_ = 2 V drain voltage was required to overcome the blocking contacts; consequently, the channel currents were measured at much lower drain voltages. Mobility in p-channel was 10^2^ lower compared to n-channel. Thus, the possibility to have high mobility in SAM-OFET molecular systems is demonstrated. These studies, along with photophysics transport studies of SAM structures, demonstrate that molecules with natural herring-bone pattern packing might be favorable for OFET applications.

### 3.2. Naphtalene Tetracarboxyldiimide (NTCDI) SAM PVC Devices

To achieve hybrid SAM-PVC, we self-assemble the NTCDA molecules, which are the same molecules used in the conductive channel of SAM-OFET, on n-type silicon substrate. The self-assembly strategy followed step-A synthetic protocol (Figure 1A). Two kind of measurement set up were used to evaluate transport in NTCDI-based SAM-PVC. The first measurement scheme relies on closed and second on open electrical circuit measurements. In the first set up, the Ag upper electrode was used to collect *photocurrects*. Semi-transparent ITO electrode was used to probe electric field created by photogenerated charges in the second set up. Combination of these measurements should (I) provide a kinetic of charge transfer in 2D NTCDI molecular system and (II) prove that this charge dynamic solely attributed to transport properties of 2D NTCDI molecular system, and not to capacitor discharge and interface charge transfer at SAM-PVC. The following section demonstrates the possibility to change the type of conductivity in 2D NTCDI molecular systems by changing substrate type from traditional n-type Si to p-type Si substrate.

#### 3.2.1. Measurements of In-Plane Currents in SAM-PVC: Closed-Circuit Electrical Measurements

**Figure 7:**
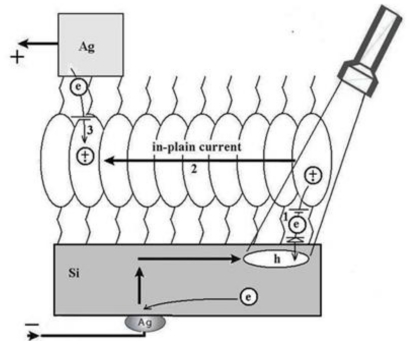
Operation of the SAM-PVC. Incident light creates holes (h) in the valence band of silicon. Upon ejection of electrons (reaction 1) from the immobilized molecules into the holes, cation radicals are formed. Cation radicals rapidly exchange with the neutral molecules (2); the positive charge travels through the film, resulting in the in-plain current. Rereduction of cation radicals by silver (3) completes the photovoltaic element. Reactions 1–3 generate electromotive force. The diode (1) reflects rapid photoejection of electrons from the film and slow back-reaction. The external electric connections used in the voltammetric experiments include a power source, an ammeter, and a voltmeter.

**Figure 8:**
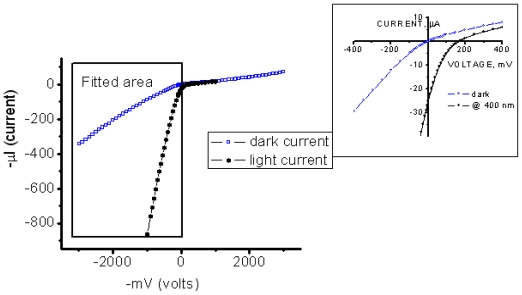
Electrooptical properties of the Si/NTCDI/Ag heterostructure. All experiments have been carried out at room temperature. Constant monochromatic 400 nm light illuminated a ~10 mm spot centered ~5 mm away from the silver electrode. When the Ag electrode is highly electronegative, the element behaves as a photoamplifier and light noticeably increases the asymmetry of the *I*–*V* curve. The marked “Fitted area” will be used to model transport mechanism in SAM NTCDI channel.

We topped the film with a small-area silver electrode and found that the resulting Ag/NTCDI monolayer/Si sandwich (Figure 7) was sensitive to light. In the dark, the current-voltage (*I*–*V*) curve goes through the origin (Figure 8) and straightens at the slopes that correspond to ~30 and ~10 kΩ, respectively, as the positive or negative voltage increases.

**Figure 9:**
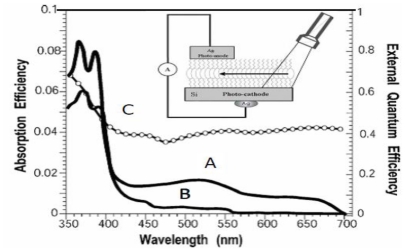
Effect of the surface density of NTCDI on the film spectra and light-harvesting efficiency. Dense film A assembled on glass has a prominent aggregation band in the greenish-orange region. The external quantum efficiency of the corresponding Si cell illuminated by constant nonsaturating light is represented by spectrum C. Sparse film B has no aggregation band in the spectrum. The external quantum efficiency of the corresponding cell was too low to measure. Insert show the measurement setup under continuous illumination.

Illumination with continuous monochromatic light changes this characteristic dramatically. Now, the *I–V* curve also goes through the bottom right quadrant where the current through the element opposes the external bias. This plainly manifests that light generates electromotive force, which induces a negative charge on silicon (photocathode) and a positive charge on silver (photoanode). The maximal photovoltage at the saturating light power was as high as 280 mV. In the absence of the film or only with the amino layer present, the maximal photovoltage was below 0.1 mV and could not be characterized accurately. The mutual arrangement of the cell and the incident light, the external quantum efficiency (EQE) of 0.4–0.7 (Figure 9), which cannot be accounted for by the absorption efficiency of the film, the dissimilarity of the EQE, and the absorption efficiency of the film and resemblance of EQE to the spectrum of silicon [71] suggest that the light is productively absorbed by the semiconductor (Figure 7).

This contrasts with light harvesting in the dye-sensitized photovoltaic cells [72,73] or the hybrid nanorod-polymer cells [74], which require special junctions with enormous contact areas between the materials to enhance their absorption efficiency. The geometry of the cell also suggests that light harvesting incorporates a longitudinal spatial energy transfer. In contrast to natural photosynthesis [75], the conserved energy does not migrate in the form of excitons. The absence of long range light harvesting, when the uncoated side of the chip was illuminated, establishes the importance of the film and rules out energy transfer within the silicon bulk.

The process starts with a redox reaction separating charges between silicon and film (reaction 1, Figure 7). Because of the spatial separation of the cathode and the anode chemistry, this reaction can be identified straight from the polarity of the cell, which shows that electrons from the NTCDI molecules are ejected into silicon, that is, NTCDI molecules oxidize. Charge separation is supported by the asymmetry of multiplication of the dark current with respect to the bias direction (Figure 8), which cannot be accounted for by the influence of nonpolar excited states. Indeed, regardless of the amplification mechanism, they would not be able to discriminate the bias directions. On the contrary, charge separation explains this asymmetry easily. Since the positive charges in the NTCDI film “reflect” in silicon “mirror”, they are accompanied by their countercharged electrostatic images and migrate as trans-surface dipoles. As these dipoles approach the Ag/film/Si junction, they are either attracted into the contact area by parallel or repelled by the antiparallel external field. In agreement with the photooxidation mechanism of energy conservation, light harvesting was observed in the thick films of *N*,*N*′-dihexyl-naphthalene tetracarboxylic diimide (C_6_-NTCDI) physadsorbed on the silicon surface, but not in the thick films of NTCDA, because NTCDA is much harder to oxidize. This photooxidation incorporates several events. Absorption of light results in elevation of an electron to the conductance band leaving a vacancy in the valence band. This vacancy can be filled either by back recombination of the electron from the conductance band (unproductive decay) or by transfer of an electron from the film into silicon (reaction 1, Figure 7) since the affinity of this vacancy for an electron is apparently high enough to oxidize a molecule of NTCDI. As a result, similarly to the photochemistry of dye-sensitized cells [76,77], an extra electron remains in the conductance band of silicon, while the oxidized molecules of NTCDI become cation radicals (Figure 7).

These radicals combine essential structural motifs of the oxidized forms of two ubiquitous redox cofactors: NAD^+^ and semiquinone. These features ease oxidation of neutral molecules. The energy of light is conserved in two forms: the electrostatic energy of trans-surface dipoles and the free energy of reduction of cation radicals by silver (reaction 3, Figure 7). The equilibrium of this reaction is shifted toward neutralization of cation radicals, and we could not attain electric currents in the films assembled on the surface of glass because measurements of such currents even over microgaps require very sensitive equipment [78].

**Figure 10:**
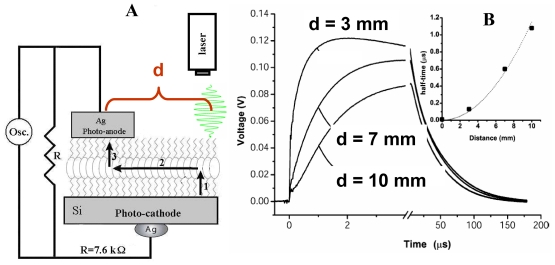
Scanning time-resolved photovoltage probing. (**A**) Measurement setup. Distance *d* is between illumination point and harvesting upper Ag electrode. The transients were induced by pulses centered at different distances apart from the silver electrode; (**B**) Kinetic of photoresponse at different *d*. Inset shows the dependency of the T_1/2_ of the voltage rise on the distance *l* between the bright spot and the silver photoanode. The solid line approximates the points by a parabola T_1/2_ = *l*^2^/*d* with the parameter *d* ≈ 10^6^ cm^2^/s.

To study migration of cation radicals to the photoanode (reaction 2, Figure 7), we used short light pulses focused at different distances from the anode to provide both time and spatial resolution. The photovoltage transients consist of voltage rise and decay (Figure 10). The decay is exponential with τ ≈ 40 μs at the given external load. This component represents the discharge of the cell because of both the backflow and the functional current through the load. The voltage rise originates from accumulation of charges at the anode and its kinetics depends on the distance between the illuminated spot and silver (Figure 10).

To avoid a model bias, we described the kinetics of the voltage rise by its half-time T_1/2_ (Figure 10, inset), which reflects the travel time of the cation radicals. Simple estimate shows that corresponding speed of charge exchange exceeds 10^4^ m/s. Despite some spatial uncertainty due to the finite beam size and a limited number of data points, one can see that the travel time appears to be proportional to the second power of the distance between the illuminated area and the silver electrode rather than to its first power. Since the area (distance quadrate) covered by a random-walking particle is proportional to its travel time, this dependency (Figure 7 (reaction 2)) is consistent with the random walk fashion of migration of cation radicals within the film during successive self-exchange reactions between the neutral molecules and the cation radicals. It is driven by the spatial gradients of their electrochemical potential, which are present because the anode reduces the cation radicals. Migration of cation radicals requires proximity of the molecules forming the monolayer and acting as redox centers. Such proximity manifests itself as a broad absorbance band in the green-orange region (Figure 9), attributed to formation of closed packed aggregate channel [62]. This migration, that is, time-resolved growth of the photovoltage, was not observed in the sparse monolayers of NTCDI, in the thick films of NTCDA, or without organic film. Only minor signals were observed in these control devices especially with the laser beam incident at the edge of the Ag contact. When the center of the beam was moved away from the edge, these signals disappeared rather than develop slower. The slight decrease of the total charge collected by the anode is probably due to partial recombination of trans-surface dipoles.

The charges formed in the remote-illuminated area have to travel within the film over “gigantic” distances at finite speed. The vast majority of studies of lateral charge transfer in monomolecular structures were limited to the self-assembling and Langmuir-Blodgett monolayers [79–81]. Some of these studies have revealed that electrochemical activity of the assembling molecules is a requirement for charge transfer within these monolayers [82,83]. In analogy to that, we believe that the efficient delivery of positive charges to the photoanode becomes possible because of the macroscopic connectivity of the network of NTCDI molecules acting as redox centers. This connectivity permits rapid transfer of large net charge through an extremely narrow cross section and originates from assembling NTCDI molecules at the positions predetermined by arrangement of the NH_2_-groups within the dense interlinked siloxane network [27].

#### 3.2.2. Measurement of Out-of-Plane Charge Recombination in SAM-PVC: Open-Circuit Electrical Measurements

When redox active molecules are covalently tethered to the surface of the n-doped silicon, light induces their oxidation by the semiconductor (see Section 2.1). One particular problem to be solved by the new method originates from our abovementioned results. In principle, the energy can be conserved for further spatial transfer in two ways. First, it can be converted into the energy of the excited state as in the photosynthetic light harvesting antennas. Second, similarly to the dye-sensitized cells, the energy can be conserved as the free energy of charge back recombination (Figures 15 and 16). In the second case, the actual case as we show, the role of the organic molecules in these SAM-PVCs is different from the role of the dye in the dye sensitized cells. The NTCDI molecules do not absorb light but act exclusively as redox couplers and form cation-radicals upon ejection of an electron into the light-induced hole in the valence band of silicon. However, distinguishing between the two possible mechanisms is challenging because adoption of the traditional methods used for the dyes-sensitized cells would make the endeavor prohibitively difficult. Switching to the IR region where silicon is transparent is demanding in terms of the equipment and still cannot help the fact that, the monolayers contain too few molecules for time-resolved spectroscopy and only a small fraction of these molecules actually forms radicals. These molecules react too fast to make single molecule techniques applicable even in principle. Finally, an attempt to produce model nanoparticles would require an adequate adaptation of the existing modification technology and there is now guarantee for the adequacy of the model system studied in the solution compared to the actual element functions in the solid state.

Experimental results of the previous section show that the diffusion time of about 1 μs or less at the given distances is apparently much shorter than the life time of the cation radicals in SAM-PVC. The similar back recombination in the dye-sensitized TiO_2_ takes milliseconds [84]. Direct measurements in the closed-circuit configuration do not allow answering the question about back charge recombination, because in this configuration a long-time part of the charge transfer kinetics depends on the input impedance of the measurement system and internal impedance of the monomolecular devise, which in turn depends on many other external parameters.

**Figure 11:**
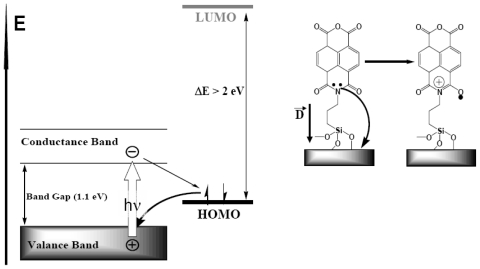
Light induced charge transfer at the silicon-NTCI(A) interface. The relative arrangement of the energy levels of silicon and NTCI(A) is necessitated by the observed polarity of the photo-induced charge transfer and by the fact that the light is absorbed by silicon. The structure of the putative cation radical is presented on the left. Vector D stands for the induced electric displacement.

Therefore, the purpose of our study became two-fold. The first goal was to find a universal and highly sensitive method to study interfacial charge transfer. The second goal was to prove that illumination of the NTCDI-modified surface of silicon results in formation of cation-radicals at the junction and thus to test the potential of the method. In order to match these goals we have opted to the surface photovoltage technique, which is sensitive to the net charge displacement [85,86], the very basic property of redox reactions. Since the spatially organized charge transfer inevitably causes electric displacement perpendicular to the junction (Figure 11), we have expected this elegant non-spectroscopic approach to be extremely useful in the studies of the surface redox chemistry.

**Figure 12:**
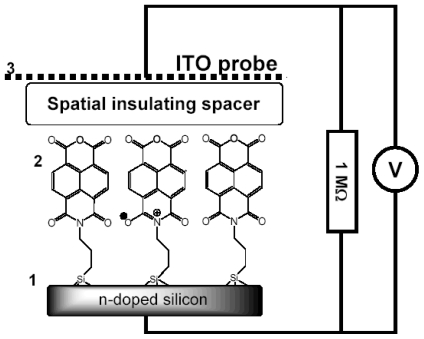
The putative surface electrochemistry and its electrostatic signature. The silicon substrate forms the capacitor plane 1. The neutral NTCDI(A) molecules and the putative cation-radical formed upon illumination form the imaginative plane 2. This organic layer is separated from the tin-doped indium oxide electrode (plane 3) by a 25 micrometer insulating film. The capacitor planes 1 and 3 are connected, respectively, to the ground and signal input of the oscilloscope (V), which has a standard input resistance of 1 MΩ.

Here we show that the charge displacement perpendicular to the surface is sufficiently strong for direct measurements. To pick this electrostatic signature, we utilize an auxiliary transparent electrode that acts as a capacitive probe (Figure 12). This probe, proposed by Bergmann in 1932, selectively picks the ac voltage [87]. Previously, the transparent auxiliary electrode has been used to study the photovoltage generated by the p-n junctions. This nondestructive no contact method proved to be convenient for the quality control of these junctions before and without equipping them with electrodes [87,88] or for measurement of the lifetimes of the minority charge carriers [89,90]. In those measurements, the ac photovoltage has been generated by a chopped light beam and its amplitude has been measured. While repetitive laser represents a limit case of the chopped beam, application of short pulses permits time-resolved measurements. Interestingly, a related approach has been utilized in the studies of the photosynthetic reaction centers in the essentially 3D membrane suspension samples [91,92].

The method measures the transient voltage drop between the plates of the capacitor formed by the semiconductor chip and a probe ITO electrode transparent to the visible light. The photovoltaic response of this device contains two components. The first one is characteristic of capacitor between doped Si and ITO electrodes and the second one is a response from organic heterojunction itself. The relaxation time constant *k* for organic heterojunction is independent on device area and identical to the constant of charge annihilation on the single molecule or cluster. That is in opposite to a capacitor response, which is area dependent.

##### 3.2.2.1. Theoretical Background for the Capacitive Probe Measurements

To proceed further, we need to develop a theory of the capacitive probe response to distinguish between the surface charge transfer dynamics and the instrumental response. Let us denote the silicon surface, the organic film and the probe electrode as planes 1, 2 and 3, respectively (see Figure 12). Charge separation between the organic film and the silicon substrate will cause formation of an electric field between the planes 1 and 2. Under uniform illumination, the concentration of the surface dipoles is the same throughout the film plane. The film becomes a surface of equal potential and can be considered as a plate of a complex 3-plate capacitor, which in turn is formed by two serial capacitors: *C*_21_ (organic film/Si) and *C*_23_ (organic film/ITO electrode). The voltage jump *U*_21_ will cause an electric current through the outer circuit formed by the probe capacitor *C*_23_, cable and internal resistance *R* of the oscilloscope. The capacities of the organic film and probe electrode were estimated to be *C*_21_ ≈ 1 μF and *C*_23_ ≈ 100 pF, respectively. Direct measurement of the cable capacity demonstrated *C* = 360 pF. Input capacity of the oscilloscope *C*_S_ = 13 pF is much smaller than the cable capacity, and in the following up calculations *C*_S_ was included into *C*.

Observed signal (voltage *U*_S_(*t*) on the oscilloscope) reflects the dynamics related to the charge exchange between capacitors and to the finite lifetime of the dipoles created in organic film and semiconductor surface. Charge annihilation in the film will cause a backward current *I*_21_(*t*) = *kQ*_21_(*t*), where *Q*_21_(*t*) is the charge accumulated in the capacitor *C*_21_, and *k* is the rate of the transient charge annihilation. As it was mentioned above, the constant *k* is defined by electrochemical properties of the sample, and does not depend on the area of illumination.

To analyze the system behavior we consider three time scales: (1) charging the organic film buy the picosecond laser pulse; (2) fast (nanosecond scale) charge redistribution between capacitors *C*_21_, *C*_23_, and *C*; (3) discharge of the system (microsecond to millisecond time scale). We assume the *C*_21_ charging event is much faster than any discharge or charge exchange process, because it is defined by the laser pulse duration ~1 ps. With this assumption, the initial voltage *U*_21_ is defined by the charge *Q*_21_^0^ generated during the light action: *U*_21_(0) = *Q*_21_^0^/*C*_21_. The initial charges of the probe capacitor *C*_23_ and the cable are supposed to be negligible: *Q*_23_(0) = 0, *Q*_C_(0) = 0.

**Figure 13:**
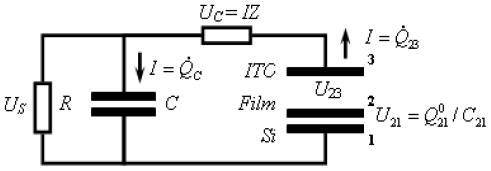
Equivalent circuit for the capacitive probe shown in Figure 12 on the nanosecond time scale.

Fast voltage jump *U*_21_ causes charge exchange in the system. Now the cable and probe electrode are being charged by the capacitor *C*_21_. At this step we take into account finite impedance of the cable *Z* = 50 Ω. The initial voltage jump is compensated by the voltage drop *U_C_* = *IZ*, where *I* is the electric current through the cable. At this time scale we can neglect the current through the oscilloscope, because its input impedance is *R* = 1 MΩ, so that *RC* ~ 400 μs, and *RC* >> *ZC* ~ 20 ns. At this relatively fast time scale of the charge exchange we also can neglect the *C*_21_ discharge and consider *U*_21_ to be constant. (We expect the typical relaxation time of transient dipoles in the organic film to be in the range of 10–100 μs, while time constants *ZC*_23_ and *ZC* are in the nanosecond range). Finally, we can describe the second step of the charge exchange by the next system of Kirchhoff’s equations according to the equivalent electric circuit shown in Figure 13:

(2) $$\begin{array}{l} U_{21}=U_{23}+U_{C}+U_{S}\\ I={\dot{Q}}_{23}={\dot{Q}}_{C} \end{array}$$

Taking into account Ohm’s low *U_C_* = *IZ*, noting that *U*_21_ = *Q*_21_^0^/*C*_21_, *U*_23_ = *Q*_23_/*C*_23_, *U_S_* = *Q_C_*/*C*, and denoting for the simplicity: *k*_23_ = 1/*ZC*_23_ and *k_C_* = 1/*ZC*, we can rewrite the Equation (1) in the next form:

(3) $$\begin{array}{l} k_{23}Q_{23}+{\dot{Q}}_{C}+k_{C}Q_{C}=\frac{Q_{21}^{0}}{C_{21}Z}\\ {\dot{Q}}_{23}-{\dot{Q}}_{C}=0 \end{array}$$

Using a Laplace transform with initial conditions *Q*_23_(0) = 0, *Q_C_*(0) = 0, we can convert the system of differential equations (3) to the system of linear equations, the latter being presented in the matrix form as follows:

(4) $$\left(\begin{array}{cc} k_{23} & s+k_{C}\\ s & -s \end{array}\right)\left(\begin{array}{c} {\tilde{Q}}_{23}\\ {\tilde{Q}}_{C} \end{array}\right)=\left(\begin{array}{c} Q_{21}^{0}/{sC}_{21}Z\\ 0 \end{array}\right)$$

Here, *Q̃*_23_(*s*)and *Q̃_C_*(*s*) are Laplace transforms of *Q*_23_(*t*) and *Q_C_*(*t*), respectively. The solution for the equation (4) is

(5) $$\left(\begin{array}{c} {\tilde{Q}}_{23}\\ {\tilde{Q}}_{C} \end{array}\right)=\left(\begin{array}{cc} \frac{1}{s+k_{C}+k_{23}} & \frac{s+k_{C}}{s(s+k_{C}+k_{23})}\\ \frac{1}{s+k_{C}+k_{23}} & \frac{-k_{23}}{s(s+k_{C}+k_{23})} \end{array}\right)\left(\begin{array}{c} \frac{Q_{21}^{0}}{{sC}_{21}Z}\\ 0 \end{array}\right)$$

It can be written in the simplified form:

(6) $${\tilde{Q}}_{23}={\tilde{Q}}_{C}=\frac{Q_{21}^{0}}{C_{21}Z}\frac{1}{s(s+k_{C}+k_{23})}$$

Applying an inverse Laplace transform to (5) we get:

(7) $$Q_{23}(t)=Q_{C}(t)=\frac{Q_{21}^{0}}{C_{21}Z(k_{C}+k_{23})}(1-e^{-(k_{C}+k_{23})t})$$

Equation (7) leads to the quite obvious conclusion. Because the charge exchange on the nanosecond time scale is controlled by the same current *I* =*Q̇*_23_ =*Q̇_C_*, the cable and probe capacitor are charged equally, and at the time scale ns the charge and voltage distribution are defined just by the capacities of the appropriate elements:

(8) $$\begin{array}{l} Q_{23}^{0}=Q_{C}^{0}=\frac{Q_{21}^{0}{CC}_{23}}{C_{21}(C+C_{23})}\\ U_{23}^{0}=\frac{Q_{21}^{0}C}{C_{21}(C+C_{23})},\;\;\;U_{C}^{0}=\frac{Q_{21}^{0}C_{23}}{C_{21}(C+C_{23})} \end{array}$$

**Figure 14:**
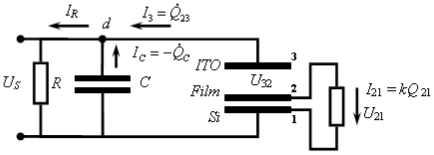
Equivalent circuit for the capacitive probe in microsecond time scale.

These values may be taken as initial conditions to evaluate the system dynamics on the microsecond time scale. The equivalent electric circuit for the measurement system at this time scale is shown in Figure 14.

Let us consider some additional approximations to simplify the final analysis. After the fast restoration of the balance in the system, we can neglect the voltage drop in the cable. The discharge of the cable is defined now by the input impedance *R* of the oscilloscope, while the cable impedance *Z* = 50 Ω is negligible compared to *R* = 1 MΩ. Another assumption is based on the fact that the faster time constant observed in experiments is in the range of 10–20 μs, which is shorter by, at least, an order of magnitude than any time constant related to the electrical discharge through *R*. This fact allows us to conclude that the discharge of the organic film is mainly caused by the electrochemical annihilation, and like in the previous case we can neglect the current to the outer circuit, so that *I*_21_ = −*Q̇*_21_ = *kQ*_21_. With these assumptions we can write the system of Kirchhoff’s equations for equivalent electric circuit presented in Figure 14 as:

(9) $$\begin{array}{l} U_{21}=U_{23}+U_{S}\\ I_{21}=-{\dot{Q}}_{21}={kQ}_{21}\\ I_{3}+I_{C}-I_{R}=0 \end{array}$$

In the last equation, which represents the currents in the node *d*, we have taken into account that in the shown configuration the current *I_C_* is discharging *C*, while *I*_3_ is charging the probe capacitor *C*_23_, so *I_C_* = −*Q̇_C_*, *I*_3_ = *Q̇*_23_, and *I_C_* =*U_S_*/*R*=*Q_C_*/*RC*. The voltages *U*_21_, *U*_23_ and *U_S_* are defined by the charges accumulated in the appropriate capacitors: *U*_23_ = *Q*_23_/*C*_23_, *U_S_* = *Q_C_*/*C*, *U*_21_ = *Q*_21_/*C*_21_. Let as also keep notation *K*_21_ = 1/*RC*_21_, *K_C_* = 1/*RC* and *K*_23_ = 1/*RC*_23_. (These constants are much lager than *k*_23_ = 1/*ZC*_23_ and *k_C_* = 1/*ZC*). Now the system (9) may be rewritten in the form:

(10) $$\begin{array}{l} K_{21}Q_{21}-K_{23}Q_{23}-K_{C}Q_{C}=0\\ {\dot{Q}}_{21}+{kQ}_{21}=0\\ -{\dot{Q}}_{23}+{\dot{Q}}_{C}+K_{C}Q_{C}=0 \end{array}$$

Applying a Laplace transform with the initial conditions (8), we convert (10) to the system of linear equations presented in the matrix form as:

(11) $$\left(\begin{array}{ccc} K_{21} & -K_{23} & -K_{C}\\ s+k & 0 & 0\\ 0 & -s & s+K_{C} \end{array}\right)\left(\begin{array}{c} {\tilde{Q}}_{21}\\ {\tilde{Q}}_{23}\\ {\tilde{Q}}_{C} \end{array}\right)=\left(\begin{array}{c} 0\\ Q_{21}^{0}\\ 0 \end{array}\right)$$

The solution of the system (11) is:

(12) $$\left(\begin{array}{c} {\tilde{Q}}_{21}\\ {\tilde{Q}}_{23}\\ {\tilde{Q}}_{C} \end{array}\right)=\frac{1}{D(s)}\left(\begin{array}{ccc} 0 & K_{23}K_{C}+s(K_{23}+K_{C}) & 0\\ \left(s+k)(s+K_{C}\right) & K_{21}(s+K_{C}) & -K_{C}(s+k)\\ -s(s+k) & K_{21}s & K_{23}(s+k) \end{array}\right)\left(\begin{array}{c} 0\\ Q_{21}^{0}\\ 0 \end{array}\right)$$

where *D*(*s*) is a discriminant of the matrix presented in (11):

(13) $$\begin{array}{l} D(s)=s^{2}(K_{23}+K_{C})+s({kK}_{23}+{kK}_{C}+k_{23}K_{C})+{kK}_{23}K_{C}\\ =(K_{23}+K_{C})(s+k)(s+\frac{K_{23}K_{C}}{K_{23}+K_{C}}) \end{array}$$

The solution for *Q̃_C_*(*s*) can be found in form:

(14) $${\tilde{Q}}_{C}=\frac{s\cdot K_{21}Q_{21}^{0}}{D(s)}=\frac{K_{21}Q_{21}^{0}}{\left(K_{23}+K_{C}\right)}\frac{s}{\left(s+k)(s+K_{\Sigma }\right)}$$

here we denote *K*_∑_ = *K*_23_*K_C_*/(*K*_23_ + *K_C_*) = 1/(*C*_23_*+C*)*R*. Applying the inverse Laplace transform to (13), we can find *Q_C_*(*t*) and the final signal *U_S_*(*t*) = *Q_C_*(*t*)/*C*:

(15) $$U_{S}(t)=\frac{K_{21}Q_{21}^{0}}{(K_{23}+K_{C})(k-K_{\Sigma })C}({ke}^{-kt}-K_{\Sigma }e^{-K_{\Sigma }t})$$

The last expression can be modified to the simplified form if we denote 1/*K*_∑_ =τ and $\frac{K_{21}Q_{21}^{0}}{(K_{23}+K_{C})C}=\frac{C_{23}}{(C_{23}+C)C_{21}}Q_{21}^{0}=U_{0}$:

(16) $$U_{S}(t)=\frac{U_{0}}{k\tau -1}(k\tau e^{-kt}-e^{-t/\tau })$$

The expression (16) shows that a monodisperse charge recombination gives rise to a bi-exponential signal. Let us consider rapid recombination of the dipoles: *k*τ >> 1. Now, it is more convenient to discuss the response (16) if it is presented in the form:

(17) $$U_{S}(t)=U_{0}e^{-kt}+\frac{U_{0}}{k\tau -1}(e^{-kt}-e^{-t/\tau })$$

The first additive term in the Equation (17) reflects the kinetics of the surface charge displacements. The second term is the deviation of the measured signal from the charge transfer dynamics. It is important that the transient crosses the *U*_S_ = 0 level before recovering the base line. Such behavior is an intrinsic property of the *RC* chain and reflects the simple fact that the probe *charges and then discharges* during the measurements, while the oscilloscope measures the *current* through its input resistance. So, the second term in Equation (17) reflects the input resistance of the oscilloscope and the capacity of the sample/probe capacitor and therefore depends on the size of the sample, length of the cable and other instrumental parameters. However, in the discussed case, *k*τ >> 1, the amplitude of the instrumental part of the response is considerably smaller than the amplitude of the signal related to charge dynamics in the sample. Therefore, the best condition for the measurements is, k 1/τ = 1/(C_23_ + C)R which may be controlled by increasing the input impedance, capacity of the probe *C*_23_ (*i.e*., by increasing the size of the surface, or decreasing the thickness of the spacer) or the cable capacity *C* (*i.e*., by increasing the length of the cable). In our experiments the instrumental time constant was in the range 0.2–2 ms.

When κτ << 1, the rapidly formed voltage *U*_0_ will dissipate as a result of charge redistribution between the plates 1 and 3 through the oscilloscope. Now, the faster component will reflect the instrumental time constant τ = *R*(*C*_21_ +*C*)<< *k*. At longer time scale the system dynamics will reflect the dynamics of charge annihilation. Because the amplitude of the first term in Equation (16) becomes small compared to the instrumental term, this configuration is not very useful for measurements. However, even in this configuration it is possible to estimate the charge annihilation rate *k*.

##### 3.2.2.2. PVC Response in Out-of-Plane Direction

The silicon chips functionalized with amino groups were prepared and left as is or topped with either sparse or dense layers of NTCI(A) as described in step-A of the Materials and Methods section. The electrometric measurements have been performed using the set up shown schematically in Figure 7, but with an uncoupled upper electrode. This electrode was a 200 nm thick ITO (indium tin-oxide) film deposited on a glass plate and separated from the NTCDI(A) film on the silicon surface by a 0.025 mm insulating spacer (Figure 12). The input impedance of the Tectronics TDS 3032 oscilloscope was set to 1 MΩ. In the experiments with an external 1:10 voltage splitter, the apparent input resistance was 10 MΩ. In these experiments, a 1/10 of the actual voltage has been applied to the input channel of the oscilloscope. The excitation light pulses at 800 nm were generated by the Ti/sapphire laser system. The pulses had 1 ps duration, 10 μJ energy (attenuated with the set of neutral density filters), and 1 kHz or 200 Hz repetition rate. Note that NTCI(A) does not absorb at 800 nm in contrast to 532 nm utilized in the experiments of the previous section. The signals were recorded by averaging of 512 oscilloscope traces. The maximum sampling rate was 2.5 GS/s.

**Figure 15:**
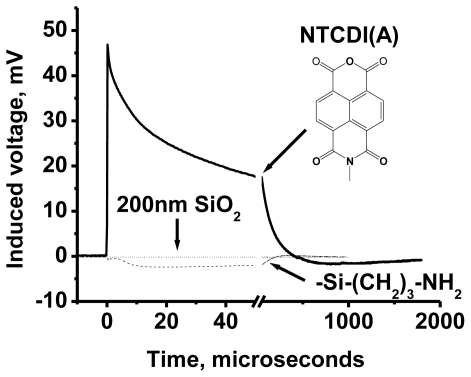
Light-induced voltage transients. Samples were used as capacitor faces separated from the ITO probe electrode by a 25 μm insulating spacer. The voltage transients represent polarization and depolarization of the Si/film junctions: Si/NTCDI(A) (solid line), Si/SiO_2_ (dotted line), Si surface derivatized with amino groups (dashed line). Here, *t* = 0 refers to the arrival time of the excitation pulse.

A typical photovoltaic signal from the sparse film of the NTCI(A) molecules is shown in Figure 15. It is complex and includes ~20 ns grouping up part, heterogeneous decay below *V* = 0 and recovery to the base line. The polarity of the signal corresponds to charging the NTCI(A) film positively. This observation perfectly agrees with our previous assumption that silicon photo-oxidizes the neutral NTCI(A) molecules, which form cation-radicals upon ejection of electrons into the semiconductor.

Minor electric responses are observed even in the absence of the NTCI(A) coating. Bare silicon surfaces are often contaminated with electrochemically active impurities. Such “as is” surfaces give highly irreproducible electric responses, which strongly decrease after rinsing the surface with acetonytrile (data not shown). This artifact is almost completely gone after NH_2_-functionalization of the silicon surface (Figure 15). The remaining signal could originate from certain polarization of the silicon waffle due to introduction of the gradient of the minority charge carriers near the surface [72]. Another possibility is the light-induced change of the built-in potential [93]. Regardless of its nature, this artifact is minor. No signal is observed when the surface of silicon is protected by a thick (>200 nm) insulating layer of SiO_2_. The insulating oxide layer prevents both the interfacial redox chemistry and band bending.

**Figure 16:**
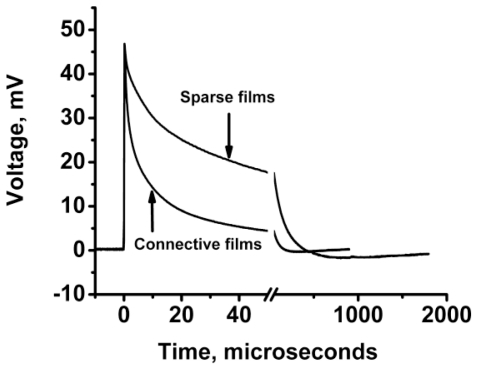
Charge recombination in connective and sparse films. Here, *t* = 0 refers to the arrival time of the excitation pulse.

While the initial charge separation is too fast to be resolved electrometrically, the heterogeneous recombination is time-resolved clearly. The fast and intermediate phases are independent of the input resistance and have a time constant of ~10 μs and ~100 μs, respectively. The contribution of the slower component into the signal varies among the samples and is about 1/3 of the total amplitude of the signal presented in Figure 16 (spare film of NTCDI(A)). The third, slowest negative component, demonstrates a clear proportionality to the input resistance and stretches 10-fold upon addition of a 9 MΩ additional resistor. Therefore, the baseline recovery likely represents the instrumental function as outlined in equations 16 and 17. This recovery component is difficult to measure because of its small amplitude.

It is important to note that in the dense NTCI(A) films the contribution of the slow (~100 μs) component of the photovoltage decay does not exceed 10% [94] (Figure 15). This observation well agrees with our previous conclusion that in the dense films the molecules rapidly exchange the oxidizing equivalent with their neighbors. Therefore, the positive charges formed in the “slow” molecules drain back to the silicon substrate via the two-step process: (i) exchange with the “rapid” molecules within connected redox network and (ii) the following discharge of these “rapid” molecules. Overall, this process is apparently faster than the direct discharge of the “slow” molecules. It is also important to note that these “slow” NTCI(A) molecules can be pre-charged in the discontinuous networks with the intense constant light [95]. A strong continuous illumination decreases the total amplitude of the signal, mainly at the expense of the slow component while the fast component remains essentially unchanged. The heterogeneity of the recombination most probably reflects the micro-heterogeneity introduced by the silicon cleaning procedures and/or the microscopically uneven thickness of the siloxane layer. Similar kinetic heterogeneity has been observed in PTCDI-doped SAMs grown on ITO by single molecule fluorescence analysis [96] and has been attributed to the differences in the local environment.

In contrast to the photosynthetic light-harvesting antennas, the energy of light is conserved by the SAM-PVC via interfacial electron transfer. The silicon/film junction forms an electrochemical capacitor charged by the light. This capacitor stores both the electrostatic energy and the free energy of the back recombination [96]. It is important to be able to measure and control the discharge characteristics of this capacitor in order to utilize it as an energy source. The electric field formed within this capacitor is picked by the auxiliary electrode and can be measured directly.

The time resolution limits of capacitive probing are defined instrumentally. A typical 2G-sample/second oscilloscope is convenient to measure charge transfers that are not much faster than ~10^8^ s^−1^. The reactions, which are slower than the instrumental time-constant, are also inconvenient to measure. Therefore, the ideal dynamic range for the capacitive measurements is between 10^3^–10^8^ s^−1^.

Capacitive probing has lower time-resolution than the pump-probe spectroscopy [85] but is free of certain limitations of the latter. First, it is independent of the optical properties of the semiconductor. Second, it works with flat surfaces, which contain much less molecules than suspensions of nanoparticles [97]. Indeed, separation of only 5.5 × 10^9^ charges per cm^2^ across ~20 Å will induce a strong enough signal with amplitude of ~1 mV (for ɛ ≈ 2). This number of molecules dissolved in 1 cm^3^ corresponds to a less than 10 pM concentration, which is far below the sensitivity limit of any spectroscopic method except for fluorescent techniques [96]. However, the electrometric approach works with non fluorescent molecules and provides a nanosecond time-resolution. The technique also provides the direction of the charge transfer.

Measurement of interface charge transfer response from ultra-fast spectroscopy is complicated in general case. The interpretation of transfer kinetics is even more challenging. For example, in well-studied Gratzell photovoltaic cells [98], the origin of two charge transfer kinetics in the femtosecond range [99] is not clear. Luckily, for SAM-PVC such delicate measurements of interface charge transfer are not required, since the kinetics of charge transport in conductive NTCDI channel (in-plane) is in the microsecond range. Nevertheless, in this scale, several components of kinetics (~10 μs and ~100 μs) have been observed. We tested few control sample devices to discriminate direct charge injection kinetics. We note that thick NTCDIA films measured with capacitor probe showed mostly long type kinetics.

This likely can be attributed to trapping of the charge the molecules, which do not have a contact with Si substrate. Exact physics of long-component response in thick NTCDI films still have to be proved unambiguously. However, it is clear that short-component of decay function (~10 μs) characterize interfacial back charge transport from NTCDI to the substrate. This is the main component in pure SAM-devices. Therefore, we can apply the developed model to determine the kinetics of charge recombination and compare with the kinetics of charge transport.

#### 3.2.3. Reversing Conductivity Channel in SAM-PVC (Closed-Circuit Measurements)

The preparation of efficient n-type conductive channel in addition to p-type conductive channel is another challenge in OFET fabrication. Moreover, it is highly desirable that n- and p-channel OFET devices are fabricated by technologically comparable methods. Mainstream research direction in OFET is attempting to develop suitable organic materials for n-type conductive OFETs. Contrary to these mainstream studies, we are trying to develop switching of the type of conductive channel based entirely on the type of substrate, which is p- or n-type silicon. The idea behind this study is the fact that “static” charge transfer self-assembled monolayers should be greatly affected by the type of substrate due to the proximity effect. Thus, different types of “Shotkey barrier”-like structures are conceivable. In turn, it could change the type of conductive channel in SAM material. To verify this assumption, we performed a photophysical study in SAM-PVC devices. The silicon chips functionalized with amino groups were prepared and topped with the dense layers of NTCI(A) on n-type and p-type silicon substrates in the same manner as described in step-A of the Materials and Methods section. Here, p-type silicon substrates (Si(100), 100 Ω·cm, Virginia Semiconductors) were tested in addition to silicon wafers of n-type (Si(100), 500 Ω·cm, Virginia Semiconductors). Both wafers had 20 Å of SiO_2_ coating. SAM-PVC devices were tested by time-resolved photovoltaic measurements in closed-circuit SAM-PVCs. Devices were illuminated at the distance 10 mm from collective electrodes.

**Figure 17:**
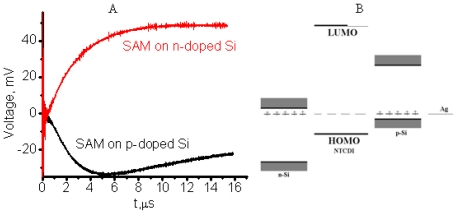
(**A**) Photoresponse of NTCDI SAM-PVC placed on n-doped and p-doped silicon wafer; (**B**) possible scheme of the molecular level, which explains switching conductivity channel in SAM-NTCDI.

The result of these measurements is shown in Figure 17. The change of the response polarity clearly indicates that the conductivity type in NTCDI conductive channels switches and depends on the conductivity type of the substrate. Difference in response kinetics is related to difference in heterostructures, doping level of Si and possible SAM density. Direct SAM-OFET testing of this idea is more complicated. Such test will require development of metal electrodes and surface chemistry to establish chemical bonds between metal and organics, which would have requested charge injection ability. Nevertheless, the described experiment could be taken as a proof of concept of such an approach. Complementary circuit design should be very straightforward once this approach will be developed for SAM-OFET. The simplicity of this approach will be due to the possibility to use the same material (as NTCDI SAM matrix) for different types of conductivity.

## 4. Discussion

### 4.1. Polaron Transport Mechanism in SAM PVC Devices

This review is focused on study of a correlation between dynamic properties and charge mobility in NTCDI SAM systems. Similar studies in bulk crystals were conducted in N. Karl’s group [55,100]. These publications reported direct time of flight measurements of charge carriers in bulk crystals of organic aromatic compound. It is remarkable that more than ten years ago, in 1998, N. Karl brought the question “Fast electronic transport in organic molecular solids?” in the title of their paper [101]. It has been demonstrated unambiguously that charge transport in the bulk crystals is intrinsic. Therefore, these researches bring a hope for the generation of scientists who developed the field of molecular electronics. In this spirit, in the beginning of this section, the question “Fast electronic transport in SAMs?” will be discussed. We have to note a difference between our experiments and the research of N. Karl’s group [55,101]. The time of flight experiments were carried out in thin organic crystals (0.2 to 1 mm thick) by applying electric field across the sample and registering the transient electric current created by illumination of the surface of the crystal by a short laser pulse. Karl *et al*. were focused on charge mobility measurements, but simple estimate shows that the speed of charges in the sample achieved 10^3^ m/s (at 300 K) to 5 × 10^4^ m/s (at 40 K) with applied electric field 20–40 kV/cm. In our experiments we did not apply longitude (in-plane) or interface (out-of-plane) biases to avoid bias polarization phenomena in SAM. In addition, our photovoltaic study was aimed to distinguish between charge transport and charge recombination processes.

Nevertheless, we have shown that in SAM the speed of charge diffusion can be as high as 10^4^ m/s at room temperature (Figure 10). One could expect that this speed may be even higher if the bias is applied along the surface. Even more surprising is the capability to initiate an electron or hole conductivity in SAM-NTCDI (Figure 17) by changing the type of doping of the Si substrate as it was observed in perylene crystal by applying different polarity of the electric field. Due to technological reasons, we could not self-assemble perelene molecules on Si (sublimation temperature of perylene tetracarboxyl dianhydride is 200 °C higher than of NTCDI). However, naphalene and perylene belong to the same class of disc-shaped aromatic compounds with extended π-electron system. Therefore, a certain analogy of transport properties is anticipated, if similar packing is achieved. It is possible to conclude that, despite of the difference between photocurrent measurements in organic crystal and SAM film, the transport phenomena are very similar and velocities of charge waves in crystal and SAM film are roughly at the same range. Moreover, the speed of charges in SAM film exceeds the same rate in organic crystal. This observation is an opposite of what could be expected from transport in 3D *vs.* 2D systems [101]. This discrepancy could be explained by role of Si substrate in NTCDI SAM/Si heterostructure, as discussed below. Due to barrier structure of this interface, out-of-plane charge transfer from NTCDI to Si could sustain in-plane current. The contribution of this mechanism is absent in crystal.

**Figure 18:**
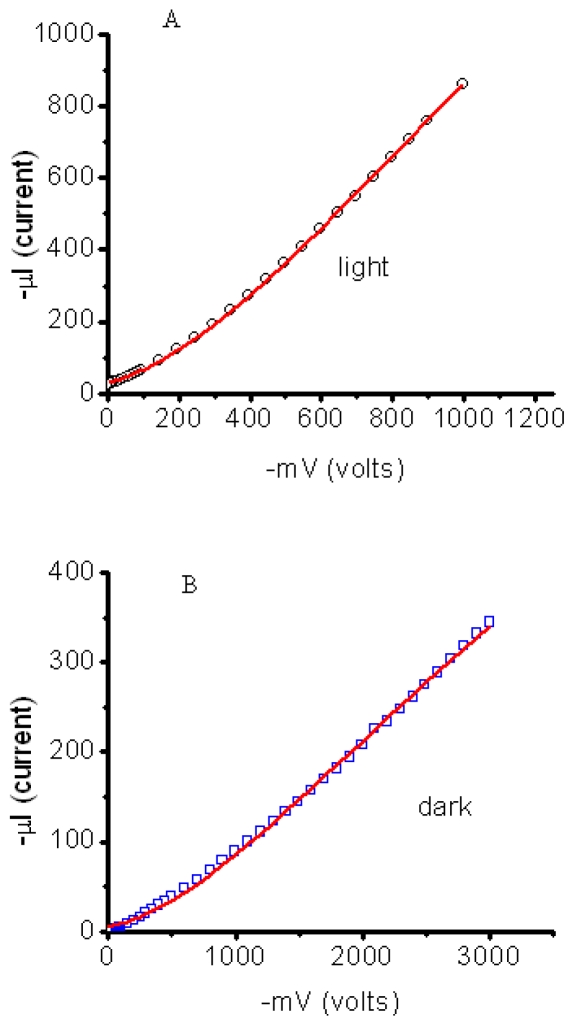
Photoresponse of NTCDI SAM-PVC, as “Fitted area” in Figure 8 and fit ln*I* ~ *E*^−2/3^ over experimental data for (**A**) photocurrent and (**B**) dark current.

Experimental results clearly indicate the possibility of polaron transport in the NTCDI-SAM 2D channel, which is located along the substrate plane. Such possibility was theoretically predicted by Horovitz [102]. He suggested that polarons dominate the photocurrent I due to a novel electric field–assisted tunneling route for which ln*I* ~ *E*^−2/3^. The negative area of our experimental SAM-PVC *I–V* (Figure 8) taken under illumination and dark conditions is presented at Figure 18 for in-plane currents. The Horovitz model (ln*I* ~ −*E*^−2/3^) is used to fit our experimental data.

We take these results as evidence of possible polaron transport mechanism in SAM-NTCDI conductive channel. Moreover, an estimate of front velocity of electronic wave in SAM-NTCDI system, (Figure 10) result in the diffusion parameter D ~ 10^4^ cm^2^/s, which is close to speed of the polaron transport. In addition, the photovoltaic studies make possible to discuss a possible type of polaron system in SAM matrix.

**Figure 19:**
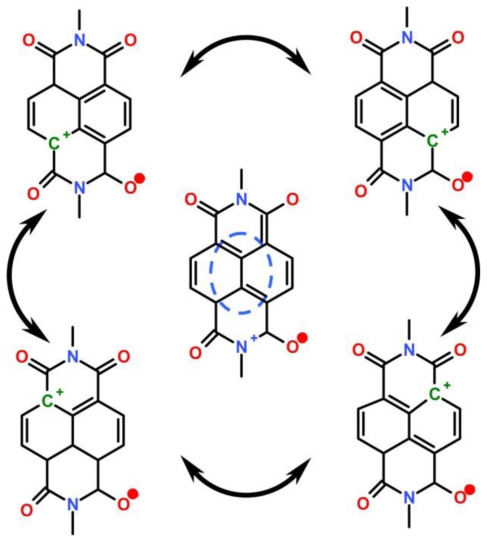
Variety of possible cation-radicals in NTCDI molecules on the molecular level. Formation of anion-radical state. Formation of cation-radical state is shown at Figure 7. This redox state could be exchanged extremely fast with neighboring neutral NTCDI molecule and transport a redox state to harvesting electrode thought self-exchange reactions. This transfer of redox state is an illustration of polaron transport in SAM-NTCDI on the nanoscale level.

**Figure 20:**
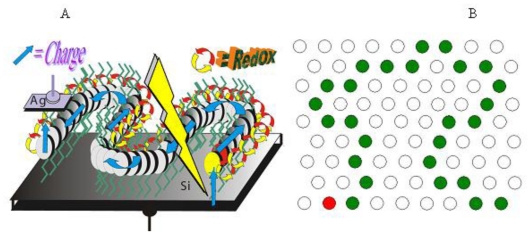
Possible cation-radicals in NTCDI molecules at the macroscopic scale. (**A**) 3D cartoon of random walk transport of redox state from illuminated area to harvesting Ag electrode; (**B**) Top view of redox transport at the macroscopic scale. Note that this system could potentially handle a large current density due to possible multi-channel transport in organic 2D system. Only one conductive channel is shown here for simplicity.

This polaron transport is likely realized in SAM-PVC device through a random walk mechanism. Excited redox state likely employs percolation-defined random walk [103], which is exemplified in Figure 19 and Figure 20.

The set of redox reactions occurring in NTCDI channel (Figure 7) includes three steps:

(18) $$\begin{array}{l} \text{1}\text{. Si}*+\text{NTCDI}\to ({\text{Si}}^{-})+({\text{NTCDI}}^{+}):\text{photo}-\text{oxidation}\\ 2.\;({\text{NTCDI}}^{+})+\text{NTCDI}\to \text{NTCDI}+({\text{NTCDI}}^{+}):\text{dark self}-\text{exchange}\\ 3.\;({\text{NTCDI}}^{+})+\text{Ag}\to \text{NTCDI}+{\text{Ag}}^{+}:\text{dark harvesting} \end{array}$$

**Figure 21:**
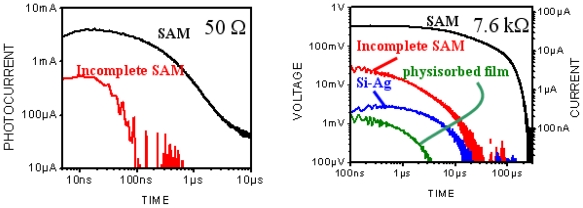
PV response in control devices with illumination at the vicinity of the contact upon different scope loads: 50 Ω (left) and 7.6 kΩ (right).

Results of photocurrent measurement in SAM-PVC could rationalize a high mobility measurement in SAM-OFETs. Indeed, assuming initiation of the same redox transport in SAM-OFET, a polaron level mobility might be expected. Assuming the polaron nature of transport in SAM-OFET, we could expect to increase the charge mobility, which currently is achieved to be as high as 31.5 cm^2^ V^−1^ s^−1^. It is a few orders of magnitude higher than in the bulk organic crystals. This mobility level could bring performance of SAM-OFET on the level of amorphous silicon FETs. This conclusion could be confirmed by photovoltaic response in control devices (defined at materials and method section), which were illuminated by light at the vicinity of contact upon different scope loads: 50 Ω and 7.6 kΩ (Figure 21). A different scope load in this case defines the time-scale and sensitivity of measurement.

In addition to SAM film, thick (~300 nm) films of NTCDA and C_6_-NTCDI were prepared by physisorption of NTCDA on the cold functionalized surface of silicon. This sample has been marked above as physisorbed film. Sparse NTCDI films were obtained from thick films of NTCDA by heat desorption of the excess of NTCDA followed by step c. This sample has been marked above as incomplete SAM. Response of Si-Ag point contact was included for comparison. Only the sample with complete SAM demonstrates a long-range charge delivery (~1 cm illumination from the electrode). The maximum voltage in SAM films exceeds that in incomplete SAM and physisorbed films by 2–3 orders of magnitude. These results clearly demonstrate that any incompleteness and disorder in SAM conductive channel affects redox self-exchange reactions on microscopic level. The time-scale of the SAM device response also demonstrates an ability of complete SAM structure to deliver charges from remote areas, in contrast to other tested devices. It is also notable that only SAM-device and incomplete-SAM-devices give rise to photo-signal at finest time scale-resolution wit 50 Ω input resistance of the oscilloscope. The fact that Si-Ag device has a higher efficiency than the device based on NTCDI-C_6_ physisorbed film is due to shielding of incoming light by a thick film.

**Figure 22:**
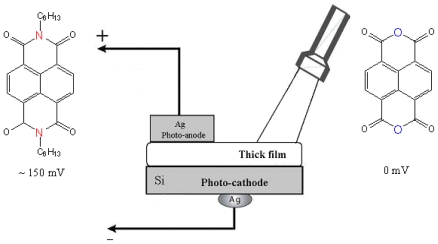
Light harvesting in thick films: molecular redox. Only film composed of C_6_-NTCDI-C_6_ molecule, which contains a nitrogen atom (red color on the left panel), was active in long-range harvesting. NTCDA molecule with unsubstantiated oxygen (blue color on the right panel) could not sustain any long-range redox transport. We took this fact as evidence of polaron transport in latent channel in SAM-PVC and SAM-OFET devices.

Transport properties of NTDCI SAM OFETs and PVCs clearly point on polaron nature of the transport. The way to prove this hypothesis is to find the way to block cation-radical exchange in the transport channel and observe change in transport properties in these devices. Experiment which addresses this possibility is shown in Figure 22. It proves the importance of redox capability to polaron transport on the molecular level. PV response of ~150 mV was observable in NTCDI-C_6_ film under illumination 3 mm away from Ag harvesting electrode, while no PV effect was observed in the case of NTCDA film. NTCDA molecules could not be oxidized at normal conditions. Thus, any un-reacted physisorbed molecular cluster will serve as an isolator for polaron transport (“redox blockade”). It is also known that OFET based on NTCDA molecules shows mobility of 10^−4^ cm^2^V^−1^s^−1^ [104], while NTCDI-based OFET routinely demonstrates mobilities, which are higher by factor 10^3^ [30–35,105]. Thus, the experiment in Figure 22 demonstrates evidence of polaron transport on the molecular level, in addition to importance of the macroscopic order. It is also notable that OFET based on NTCDA molecules [105] shows mobility of 10^−4^ cm^2^ V^−1^ s^−1^, while NTCDI-based OFET [105] routinely demonstrates mobilities which are by a factor of 10^3^ higher. Therefore, “redox blockade” experimental (Figure 22) and literature data [30–35,105] prove the polaron transport in the NTCDI-SAM channel. Thus, any unreacted physisorbed molecular cluster will serve as an isolator and trap for polaron transport.

Polaron conductive mechanism could explain why at the current stage it is so difficult to achieve high mobility in SAM devices. Both macroscopic and microscopic orderings are important for polaron transport and this imposes strict conditions on the quality of SAM conductive channel. Taking into account short range of dipole-dipole interaction, any defect could trap redox state. In addition to that, trapped redox state will form stable charged trap, which will prevent transport not only through the defect point, but also in the vicinity of this defect. Trapped redox state should affect the efficiency of charge transfer more than existence of non-reactive molecular cluster, which not carrying any charge. As the number of such trap exceeds percolation limit, no polaron transport will exist in such a system. Thus, in some analogy to biological systems, a redox active path in SAM matrix and good packing are the pre-condition for an efficient charge transport in the SAM systems.

Based on this assumption, we suggest that switching of conductivity type is possible in conductive channel. It is due to the fact that (i) various forms of cation-radical are possible and (ii) proximity of interface with inorganic semiconductor. Formed anion- and cation radicals, as shown in Figure 17, correspond to p- and n-type conductive channel in OFET. Our SAM-PVC measurements indicate that changing doping of substrate could indeed change the current sign and, thus, the type of conductive channel in SAM-PVC. Although this assumption should be confirmed in SAM-OFET system, our observation could result in alternative way to achieve efficient OFETs with tunable type of conductive channel. Moreover, one could conceive the cascade FET devices, which would have variable mobility in conductive channel according to selective doping of substrate.

**Figure 23:**
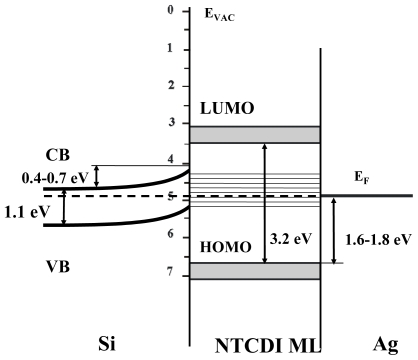
Energy diagram of the heterostructure of 2D NTCDI transport channel according to SAM-PVC studies. Energy levels inside HOMO-LUMO gap are shown by thin lines inside NTCDI monolayer (ML). These energy levels appear due to charge delocalization inside NTCDI ML molecular aggregates.

Finally, we could summarize results of our SAM-PVC and reported studies [106,107] with an energy diagram, which shows positions of the energy levels at the heterostructure of p-type 2D NTCDI transport channel, in Figure 23. The left part of this diagram shows conductive (CB) and valence (VB) bands in silicon substrate and slope of the bands as they approach the interface with SAM NTCDI. The middle panel demonstrates HOMO-LUMO (HOMO = highest occupied molecular orbital, LUMO = lowest occupied molecular orbital) gap and COB (COB = continuum of band, [108]) of NTCDI monolayer. The right part of the diagram shows the position of Fermi level *E*_F_ of Ag electrode.

The Si/NTCDI interface of SAM-PVC (left part of Figure 23) models the interface of conductive channel on silicon in SAM-OFET device. In this interface, the out-of plane photo-oxidation of NTSDI takes place. The structure of NTCDI SAM is modeled in the middle part of the diagram. This part is responsible for the charge transport in SAM-OFET NTCDI 2D system. A set of thin lines in the vicinity of *E*_F_ shows continuous of band (COB) of 2D NTCDI system, which is also called sometimes “the-states-in-the gap”. Transport through COB is in-plane dark self-exchange step in Equation 18. Note that the SAM-PVC device does not have direct access to HOMO-LUMO levels, nor SAM-OFET. Of course, molecular orbitals essentially change their distribution as the redox goes through the molecule, but charges do not have to overcome energy barriers, which involve molecular LUMO orbitals. Boundaries of molecular stacks are the only places where tunneling is governing the transport mechanism [109–111]. The right panel of Figure 23 shows the Fermi level of the electrode inside the COB. The possibility of such position of *E*_F_ was proven in [112,113]. This part of SAM-PVC system corresponds to out-of-plane dark harvesting (Equation 18) and should model charge injection into NTCDI channel from Au electrodes in SAM-OFETs. Figure 23 represent the “static” model which describe SAM heterostructure in neutral manifold. While the polarons are propagating along the in-plane COB channel, the out-of-plane electronic “pump” (reaction 1 in Figure 7) could provide an electrical motion force for the charge-delivery. This contribution could explain the higher charge speed in SAM layer than in organic crystal.

Therefore, the charge kinetic studies described above demonstrate that the out-of-plane charge injection can be discriminated. This discrimination is possible through illumination of the SAM-PVC device in different positions. Thus, variable kinetics of in-plane charge transport could be separated from the constant value of charge injection. The whole balance of device kinetics can now be visualized and used for optimization of the SAM-OFET design. Our studies underline the importance of careful comparison of the electrical and optical data to discuss the major transport mechanism in SAM system. The photocurrent study was the only way to split and estimate efficiency and performance of different SAM PVC components, such as in-plane conductive channel and out-of-plane charge separation and injection. Using optic and electronic studies we were able to evaluate the major transport mechanism in NTCDI-based SAM-PVC. A complex interplay of the molecular properties and solid-state properties was highlighted by SAM-PVC studies in NTCDI conductive channels.

There are questions about the optimal structure of conductive channel in the SAM-PVC, which include determination of what kind of crystal packing (e.g., herringbone *versus* π-stacking) is most favorable for the charge transport and whether intermolecular electronic cloud overlap can be enhanced by tuning the crystal packing (e.g., the degree of intermolecular ring slip) or by increasing the strength of intermolecular bonding [112]. Our results indicate that high charge mobility could be achieved in herringbone packing molecules, for example NTCDI molecules, which have one of the highest mobilities for organic molecules. Significantly, closing the structure-property knowledge gap on low-dimensional phase transitions in organic solids [109] is a required component to get insight into structure-property relationship of SAM-PVCs and other organic devices with epitaxial order.

We also want to note that SAM-PVC was used without any protection layer and demonstrated good device stability, which is perhaps one of the most crucial issues in organic PVC. SAM-PVCs were studied in an operational regime for two weeks without any protection from the ambient. Two-week check of SAM-PVC did not show any decrease of the semiconductor sensitivity due to O_2_ and H_2_O. The *I–V* characteristics did not show a hysteresis as a function of device operating parameters. This result agrees with previous results on OFET performance based on functionalized NTCDI system, such as NTCDI-CH_2_C_6_H_4_CF_3_ [4]. These structures offer the best combination of high mobility and air stability.

### 4.2. Model Compounds

#### 4.2.1. Structure of Model Compounds and Liquid Crystals (LCs)

The model compounds are designated as low weight organic compounds, which have the same structural NTCDI core moiety as VP-SAM films. Therefore, these compounds are suitable to be used to model the structure and properties of VP-SAM devices. The study of solid state VP-SAM films requires delicate analytical measurements. This is due to the nanoscale thickness of VP-SAM films and the low electron density in organic compounds. In deference to that, the model compounds are organic solids, which are available in macroscopic state (*i.e*., crystals) and soluble in most of organic solvents. Thus, these compounds are convenient for standard XRD, analytical chemistry and photo-physical measurements. The study of model compounds should clarify the interconnection between structure, optical and charge transport properties, as well as exploring charge delocalization phenomena in solid state VP-SAM films in more details.

We designated organic chains that were attached to both sides of the NTCDI monolayer molecular core as spacers. Two model compounds were synthesized using short spacers: aliphatic *N*,*N*′-dihexyl-NTCDI (**1**) and aromatic *N*,*N*′-diphenyl-NTCDI (**2**) compounds. The physical dimensions of compounds **1** and **2** are comparable with the size of NTCDI moiety in VP-SAM films. Additionally, the model compounds with the longer spacers aliphatic *N*,*N*′-xehadecyl-NTCDI (**3**) and aromatic *N*,*N*′-diphenyl-pyridin-NTCDI (**4**) compounds were studied to verify the trend in solid state in-plane packing in SAM films, which is expressed in the ability of the model compounds to form liquid crystals (LCs). A photoluminescence (PL) of aliphatic *N*,*N*′-dihexyl-NTCDI (**1**) compound was investigated in its single crystal form. The packing and photophysical properties of the model compounds were correlated with the electric field dependent electroluminescence (EL) [37], structure-tunable emission and virtue of in-plane polarons in solid state organic structures. All model compounds have a LC ordering, which is the manifestation of in-plane packing in VP-SAM films. This explains the strong aggregation tendency observed in VP-SAM structures [61,62].

LCs were tried as active components in electroluminescent devices, molecular wires and fibers, photorefractive materials, photovoltaic cells, and other media for high-information-density [115,116]. An aromatic *N*,*N*′-diphenyl-NTCDI has an ionization threshold of 7.4 eV [117]. This value of the ionization threshold is comparable to that of tetracyanoquinoinodimethane, which is widely used with charge transfer complexes. This high ionization threshold is a result of the inductive effect of four-carbonyl oxygen as observed in other organic materials with multiple carbonyl oxygen atoms [118]. Naphthalene tetracarboxyl diimide (NTCDI) was tried for blue light-emitting organic electroluminescent devices along with *N*-alkyl or arenyl-4-acetylamide 1,8-naphtalimides [119]. NTCDI, a low weight compound, was found to form exciplexes with diamine in the solid state, resulting in a long-wavelength PL emission due to the exciplexe [120]. *N*,*N*′-bis(octyl)-NTCDI was used as a doping material for organic photovoltaic cells [121]. This type of doping in a poly[2-methoxy-5-(2′-ethylhexyloxy-*p*-phenylvinylene) (MEN-PPV) matrix set in Scotty-type device increased the current density and circuit voltage by a factor of 10 and 6, respectively. The *N*,*N*′-dihexyl-NTCDI was employed in photorefractive applications [122]. The LC-like structural transformations that were observed in conjugated *N*,*N*′-bis(2-phenyl)perylene-3,4,9,10- bis(dicarboximide) were used for fabrication of optical discs [123]. The photochemical response in naphthalimides and naphthaliimides has been tried for multipurpose applications in biological systems [124].

#### 4.2.2. Tracing Imide Bonds in Model Compounds

**Figure 24:**
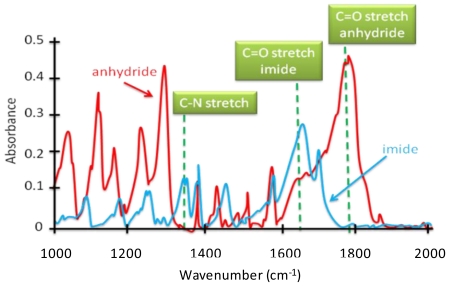
FTIR spectra of NTCDA, VP-SAM precursor *vs.* NTCDI imide model compound **1**. Apparent vibrations in imide model compound point to the formation of imide bridge.

Tracing the formation of covalent bonds in self-assembled films (imide bonds in the case of NTCDI SAM films) is imperative to insure its structural integrity. Weaker van der Waals bonds between adjacent organic monolayers in SAM structure would result in a discrepancy between intended and actual SAM structure. The direct identification of covalent bonds in SAM film is an accessible measurement [108], however its feasibility still has to be established rigorously in different molecular systems. It follows that tracing covalent bonds in model compounds might be useful in verifying formation of covalent bonds in SAM films. The formation of imide bonds between diamino-compounds and 1,4,5,8-Naphthalene-tetracarboxylic dianhydride (NTCDA) VP-SAM precursor are validated by FTIR spectroscopy of the model compound **1** as depicted in Figure 24. UV-Vis and FTIR ν_C=O_ spectral bands of NTCDA precursor have maxima at 369 nm and 1779 cm^−1^.

These UV-Vis and FTIR ν_C=O_ spectral bands were shifted from 369 nm to 381 nm and from 1779 cm^−1^ to 1655.9 cm^−1^ for compound **1**. The spectral bands were shifted from 369 nm to 380 nm and from 1779 cm^−1^ to 1655.5 cm^−1^ for compound **2**. Correspondent shifts were observed from 369 nm to 382 nm and from 1779 cm^−1^ to 1655.7 cm^−1^ for compound **3**. Eventually the shifts from 369 nm to 379 nm and from 1779 cm^−1^ 1655.8 cm^−1^ were observed for compound **4**. These shifts were due to formation of covalent imide bonds between NTCDI-core and spacers in model compounds. The surface condensation reaction between alkylamine and NTCDA studied by FTIR measurements of alkyl-amine monolayer self-assembled on Au/SiO_2_/Si substrate. The alkyl-amine peak at 3200 cm^−1^ disappeared and the imide peak at 1655 cm^−1^ appeared after the reaction with NTCDA in MLE-derived films on gold. The comparison of FTIR spectra of NTCDA VP-SAM precursor, model **1** and VP-SAM films provide evidence of the formation of imide bonds in the VP-SAM structure.

#### 4.2.3. Phase Properties of Model Compounds

**Figure 25:**
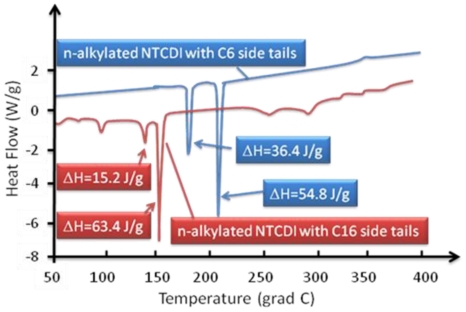
Differential Scanning Calorimetry (DSC) of model compounds **1** (C_6_) and **3** (C_16_). The heat direction is concise with a growth of heat flow scale.

LC properties of the model compounds were studied by Differential Scanning Calorimetry (DSC) and observations of the optical textures on a polarizing microscope equipped with a hot-stage. Calorimetric data for compounds **1** and **3** are shown in Figure 25. DSC traces of the reactive precursor mixtures of NTCDA + DAH (diaminohexane) and NTCDA + MDA (methylene dianiline) were presented earlier [125]. Compounds **1** and **3** are in LC phase over a large temperature range. The LC phase for the model compound **4** was observed up to T = 220 °C. Similar LC phase were observed at a wide temperature range in dioxotriazole derivatives of perylene [114]. This is likely due to the similar polyaromatic core of these compounds. We note that LC→Ps transition could not be observed in the XRD spectra for the model compound **1**. It seems likely that the LC transition in compound **1** may be obscured by the high viscosity present in the LC phase [121]. The minimum requirements for a molecular structure to produce the LC phase are not yet clear. However, it is known that for porphyrins and phthalocyanines, which have aromatic cores slightly larger than the NTCDI-core, eight side chains are required to induce LC. For example, in LC zinc octakis-(octylethyl ether) porphyrin [114], the ratio of side chain atoms (excluding hydrogen) to atoms in aromatic core is 3.6, whereas for **1**, **2**, **3** and **4** the ratio is 0.6, 0.7, 1.6 and 0.8, respectively.

DSC thermograms for compounds **1**, **3** and **4** indicated at least a two-step melting process, which points to temperature dependence of ordering processes in model compounds. The DSC dependences of **2** do not exhibit LC transition. Two sharp bands were observed in the area of 200 °C for compound **1** with a dihexyl spacer. The model compound **3**, which has a long xehadecyl chain, was closer to traditional liquid crystal and exhibited a multiple peak structure. However, two strong peaks were clearly apparent in the DSC spectra of compounds **1** and **3**. The perylene-core compounds, such as *N*,*N*′-bis(2-phenyl)perylene-3,4,9,10-bis(dicarboximide) [121] have a very similar features to **1** and **3**; the naphatlene-core model compounds. The difference is seen in a higher LC transition temperature of perylene-core compound, which is shifted to 300 °C in the case of *N*,*N*′-bis(2-phenyl)perylene- 3,4,9,10-bis(dicarboximide) [121]. This is not surprising since the perylene-core is roughly twice as large as the naphatlene-core. Hence, we would expect stronger van der Waals interaction in the perylene-core compounds and higher LC transition temperature. The phase transitions in the model compounds (depicted in Figure 25) point to the possibility of in-plane ordering in VP-SAM structures and explains structural stability in these SAM films. Formation of the transient phase in the model compounds **1** and **3** was apparent in polarized optical images and XRD spectra.

**Figure 26:**
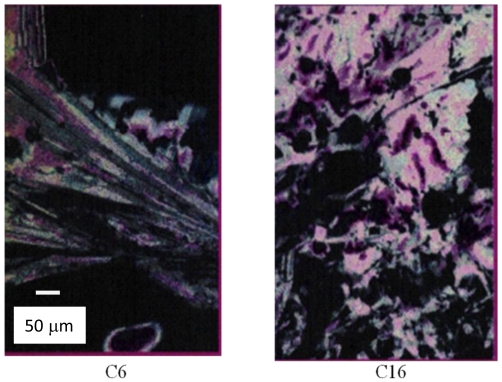
Polarized optical image of model compounds **1** (left) and **3** (right) at 225 °C and 160 °C correspondingly. Mesophases of N-alkylated NTCDI model compounds verified by polarizing optical microscopy. The ruler bar for the right image is the same as for the left image.

DSC of compounds **1** and **3** (Figure 25) indicated presence of more than two phases. These phases have been observed by polarized microscopy within a temperature range from room temperature until above melting point as shown in Figure 26. Compounds **1** and **3** are designated as C_6_ and C_16_ correspondingly, according to the length of aliphatic chains, which were attached to the aromatic core. In this experiment, the temperature of the substrate (and presumably the sample) was kept close to the transition point, 225 °C and 160 °C for the model compounds **1** and **3**, respectively. These acquired pictures are similar to typical images of mosaic liquid crystals, which show a certain crystallographic order even after melting.

**Figure 27:**
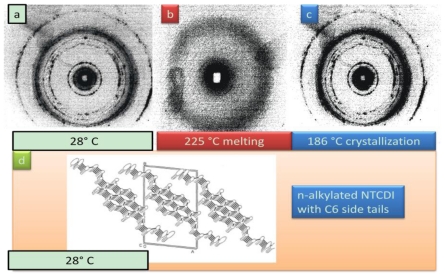
Temperature dependent XRD spectra of model compound **1** revealing the strong tendency to face-to-face stacking in smecktic phase at (**a**) room temperature (T = 28 °C), (**b**) melting (T = 225 °C) and (**c**) recrystallization (T = 186 °C). (**d**) Highlight of a crystal structure of **1**. XRD spectra in (a) and (c) are similar. Above the melting point the crystalline pattern vanished in part and became diffuse as it appears in (b), due to loss of a long range order.

LCs possess the remarkable ability to self-organize spontaneously into highly ordered structures that, when disturbed, simply restore the original structure. Temperature dependence of XRD spectra of compound **1**, revealing the strong tendency to face-to-face stacking even during melting, is shown in Figure 27. At room temperature (Figure 27a, T = 28 °C) the (n00), *n* = 1–3 and (201/−112) planes dominate the structure [122]. At melting temperature (Figure 27b, *T* = 225 °C) two spread singlets of (100) and (200) are still present. After the cooling cycle at a temperature of 186 °C (Figure 27c), the growth of (n00) and (003) orientation is observed. After re-crystallization, there is a noticeable growth of crystal ordering in the compound, the integral intensity of (n00) peaks (Figure 27c) rose by more than 10-fold in comparison with the (n00) peaks of the XRD spectrum in Figure 27a.

The temperature dependences of XRD spectra indicate the self-ordering processes of the model compounds. The extended stability in melting condition seems to be connected to a strong tendency for aggregation in heterocyclic compounds. The observed structural stability of the model compounds is due to phase-to-phase aggregation.

#### 4.2.4. Analogy of π-Aggregation in Model Compounds and VP-SAM Structures

**Figure 28:**
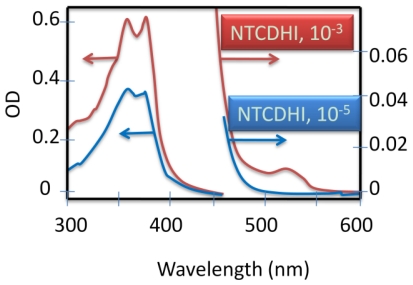
UV-Vis absorption spectra of aliphatic **1** model compounds at 10^−5^ M and 10^−3^ M in DMF. The 300–450 nm range corresponds to the isolated state, while π-aggregation leads to formation of 480–600 nm bands. Similar bands of “isolated” and π-stacked molecules have been observed in solid state VP-SAM structures.

Formation of new π-stacked bands in solutions of the model compounds was studied by absorption and photoluminescence (PL) spectroscopy. At low concentration (10^−5^ M), only visible bands of monomers were present in the absorption spectrum. At high concentration (10^−3^ M), growth of monomer peak intensity in blue region was observed. At the same time, the band attributed to formation of π-stacked molecular aggregates was observed in the red (Figure 28).

The blue absorption and PL peaks were attributed to radiate recombination on a single NTCDI molecule while the red absorption and PL were assigned to a radiate recombination within NTCDI molecules. The coexistence of two emitting centers within the same layer is an indication for two different redox paths: (a) intra-molecular redox and (b) inter-molecular redox. These two emission centers could be clearly separated in the spectroscopic study of model compounds.

**Figure 29:**
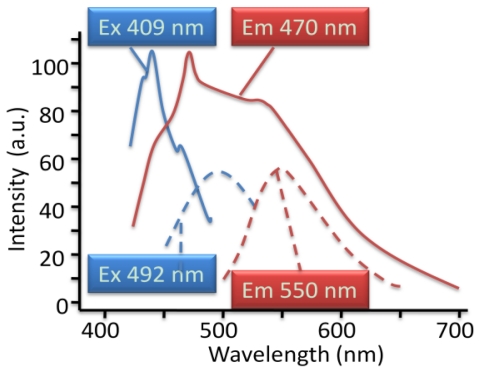
Luminescence spectra of model compound **4 (**NTCDI-bpy) in anisole, 0.267 μM in isolated state (solid lines) and exhibiting π-aggregation (dashed lines).

The emission and excitation PL spectra of the model compound **4** are shown in Figure 29. Two different peaks of PL were clearly observed. One band was located in the region 400–430 nm and a second broad peak was located at about 570 nm. Those bands were attributed to monomer and aggregate PL, respectively.

**Figure 30:**
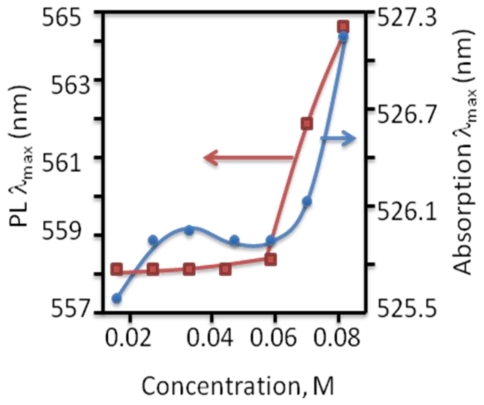
Concentration dependent linear optical properties of photoluminescence (PL) in the visible area: blue shifted PL (excitation 230 nm) and absorption spectra of NTCDI-HM in chloroform.

Experiments with compound **1** reveal the clear concentration dependence of PL and absorption spectra as it is depicted in Figure 30. The general trend is blueshift absorption at lower concentration.

**Figure 31:**
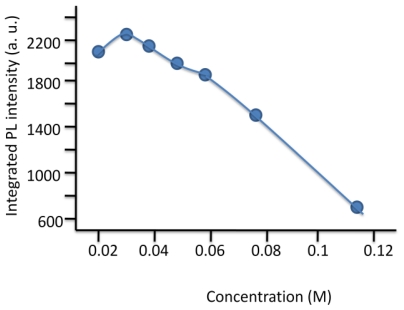
Concentration dependent linear optical properties of **1** in visible area: integrated photoluminescence (PL) intensity (excitation at 230 nm) of NTCDI-HM in chloroform.

The local maximum at low concentration can be explained by formation of *h*- and *g-*aggregates. The dimmers are supposed to dominate in concentrated solution at this concentration. PL of compound **1** decreased with molecular concentration, as shown in Figure 31. It is luckily due to energy transfer from radiate intramolecular excitation to non-radiate intermolecular excitations. Indeed, the possibility of relaxation inside the molecular aggregate became much larger as the molecular aggregates became the dominant feature at higher concentrations.

**Figure 32:**
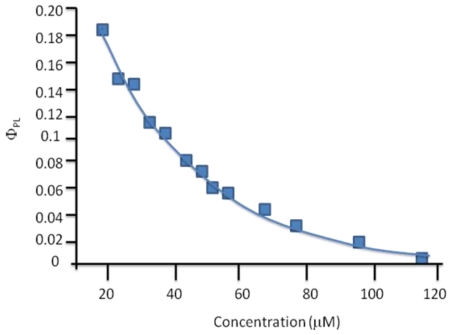
Concentration dependence of photoluminescence (PL) quantum yield of compound **1** in the visible area (excitation 230 nm and emission 560 nm).

Decrease of integrated PL intensity with increasing of the concentration followed the similar trend in concentration dependence of PL quantum yield. The concentration dependence of the quantum yield, as shown in Figure 32, exhibited the essential PL quenching with increase of the concentration.

The correlation between the PL, electrical field dependent electroluminescence and a strong tendency of model compounds to aggregate has been reported [126].

This study of model compounds clarifies a critical dependence of packing and the electronic environment in conductive channels on the performance of SAM PVC. Indeed, attachment of different spacers to the NTCDI core could not be considered as doping in inorganic semiconductor materials. However any changes, such as different spacers, could cause the major change in RedOx transport, due to various proximity effects. Therefore, a minor change in electronic environment could cause essential changes in transport mechanism. This observation is very similar to a spacer dependent property in a bio-molecular system [127]. Moreover, a polaron transport over the molecular aggregate should be taken into account along with RedOx process inside the molecules (Figures 19 and 20). These considerations could serve as a roadmap for future research. Experiments with cation-radical blockade (Figure 22), were based on comparison of photovoltaic responses between the naphthalene tetracarboxyl dianhydride (NTCDA) precursor material and tetracarboxyl diimide (NTCDI) model compound. Studying model compounds provided critical evidence verifying the polaron nature of transport in VP-SAM devices.

#### 4.2.5. Weak Delocalization in SAMs and Model Compounds

A quantum mechanical concept often applied in organic chemistry to describe the π-bonding in a conjugated system is that this bonding is not localized between two atoms: instead, each link has a “fractional double bond character” or bond order. There is a corresponding “delocalization energy”, identifiable by the stabilization of the system compared to a hypothetical alternative in which formal (localized) single and double bonds are present. Some degree of delocalization is always present and can be estimated by quantum mechanical calculations. The effects are particularly evident in aromatic systems and in symmetrical molecular entities in which a lone pair of electrons or a vacant π-orbital is conjugated with a double bond. Delocalization in such species may be represented by partial bonds or as resonance between contributing structures.

The charge delocalization in organics are quite different from delocalization in inorganic media, such as in inorganic metals and semiconductors. The textbook example of charge delocalization in inorganic materials leads to hybridization of electronic orbitals due to principles of quantum mechanics. This hybridization is results in formation of conductive and valence bands. Solid state physics allows for an electron to be considered as a free particle inside these zones.

The overlap of electronic orbital in organic solids usually is not as strong when compared to inorganic semiconductors or metals, and therefore wide energy bands for mobile charges can not be formed. The opportunity to exchange charges between neighboring molecules defines the polaron-assisted type of charge delocalization in organic material; even in the very narrow bands that are present in organic materials. This illustrates the concept of polaron in organics as a mobile molecular condition that exhibits some type of redox state that transfers faster than charges. It is a relatively new idea for solid state physics. Accounting for the many-body phenomena in molecular transport at the nanoscale level is another example that distinguishes transport in organic from inorganic structures [128–130].

An insight of delocalization phenomena and their contribution to transport mechanisms in organics still remains to be achieved. A remarkable feature of these model compounds is their capability to reproduce, or even underline, the in-plane packing in VP-SAM structures. In this context, the study of model compounds was a necessary first step toward the molecular engineering of VP-SAM devices.

The fabrication of nanodimensional organic media with a predicable structure, applicable characteristics and knowable physics are critical steps for the development of molecular nano-electronic devices.

## 5. Conclusion

This review highlights various problems and challenges that we are facing during development of nanoscale molecular photovoltaic devices. We have demonstrated that the development of adequate analytical tools, combining electric and opto-electronic studies, is crucial for future progress in SAM-PVCs. In the current state-of-the-art, a variety of Kelvin probe-derived techniques are widely used to determine electron work functions and bend banding in organic-inorganic heterostructures. UV and inverse photoemission spectroscopy (UPS, IPES) are used to determine the position of the highest occupied and lowest unoccupied molecular orbitals. XPS measurements serve to estimate band bending. Comparison of these techniques with measured *I*–*V* characteristics enables one to determine the mechanism(s) that control charge transfer in molecular diodes. These methods are static, since probing interfaces in time-scale compared to interface charge transfer is very challenging. Ultra-fast spectroscopy was applied to clarify dynamics of charge transfer in organics. Nevertheless, direct observation of the complete dynamics of charge transfer in the interface is currently beyond the scope of these methods. We suggest the use of photocurrent measurements in a time-resolved regime as a new tool, supplementary to direct PVC measurements to probe hybrid molecular interfaces. The possibility of studying charge transfer kinetics demonstrates that the charge of the out-of-plane injection and in-plane transport in an organic channel pathway can be discriminated. Thus, a new photocurrent approach can be an important analytical tool for the molecular engineering of SAM PVCs. The possibility to evaluate and compare the rate of charge injection and charge transport in these hybrid devices to achieve optimal device performance is undeniable. The application of photophysics studies of SAM devices makes possible the analysis of transport properties in organic systems and will provide future insight into charge transfer in nano-dimensional objects.

Investigation of transport mechanism in NTCDI-based SAM-PVC by means of photoelectrical measurements demonstrates that a moderately high, and perhaps greater mobility can be achieved. The achieved mobility in NTCDI SAM devices is roughly 10-times larger than the value reported for NTCDI thick film [30–35]. This improvement in the device’s performance would be achieved by better packing in PVC devices and better conditions for redox exchange transport, as is evident from structure-photoresponse study. Experiments with cation-radical blockade and conductivity switching in photovoltaic devices should be employed in the development of a new vision for PVC devices, based upon the molecular self-assembly approach.

This review highlights an example of a molecular engineering approach, in which a charge delocalization through molecular stack should not only be not neglected, but must be further explored. Our analysis of the electron transport mechanism in an SAM-PVC system clearly demonstrates the need for further research into this most interesting field. The theoretical foundation of cation-radical polaron transport in organic systems with a weak delocalization is wide open for investigation and development.
